# Prior dengue virus serotype 3 infection modulates subsequent plasmablast responses to Zika virus infection in rhesus macaques

**DOI:** 10.1128/mbio.03160-23

**Published:** 2024-02-13

**Authors:** Tulika Singh, Itzayana G. Miller, Sravani Venkatayogi, Helen Webster, Holly J. Heimsath, Josh A. Eudailey, Dawn M. Dudley, Amit Kumar, Riley J. Mangan, Amelia Thein, Matthew T. Aliota, Christina M. Newman, Mariel S. Mohns, Meghan E. Breitbach, Madison Berry, Thomas C. Friedrich, Kevin Wiehe, David H. O'Connor, Sallie R. Permar

**Affiliations:** 1 Human Vaccine Institute, School of Medicine, Duke University, Durham, North Carolina, USA; 2 Division of Infectious Disease and Vaccinology, School of Public Health, University of California, Berkeley, California, USA; 3 Department of Pediatrics, Weill Cornell Medicine, New York, USA; 4 Department of Pathology and Laboratory Medicine, University of Wisconsin-Madison, Madison, Wisconsin, USA; 5 Department of Veterinary and Biomedical Sciences, University of Minnesota, Twin Cities, St. Paul, Minnesota, USA; 6 Department of Pathobiological Sciences, University of Wisconsin-Madison, Madison, Wisconsin, USA; Dartmouth College, Hanover, USA

**Keywords:** Zika virus, dengue virus, plasmablast, B cell, neutralizing antibodies

## Abstract

Immunodominant and highly conserved flavivirus envelope proteins can trigger cross-reactive IgG antibodies against related flaviviruses, which shapes subsequent protection or disease severity. This study examined how prior dengue serotype 3 (DENV-3) infection affects subsequent Zika virus (ZIKV) plasmablast responses in rhesus macaques (*n* = 4). We found that prior DENV-3 infection was not associated with diminished ZIKV-neutralizing antibodies or magnitude of plasmablast activation. Rather, characterization of 363 plasmablasts and their derivative 177 monoclonal antibody supernatants from acute ZIKV infection revealed that prior DENV-3 infection was associated with a differential isotype distribution toward IgG, lower somatic hypermutation, and lesser B cell receptor variable gene diversity as compared with repeat ZIKV challenge. We did not find long-lasting DENV-3 cross-reactive IgG after a ZIKV infection but did find persistent ZIKV-binding cross-reactive IgG after a DENV-3 infection, suggesting non-reciprocal cross-reactive immunity. Infection with ZIKV after DENV-3 boosted pre-existing DENV-3-neutralizing antibodies by two- to threefold, demonstrating immune imprinting. These findings suggest that the order of DENV and ZIKV infections has impact on the quality of early B cell immunity which has implications for optimal immunization strategies.

**IMPORTANCE:**

The Zika virus epidemic of 2015–2016 in the Americas revealed that this mosquito-transmitted virus could be congenitally transmitted during pregnancy and cause birth defects in newborns. Currently, there are no interventions to mitigate this disease and Zika virus is likely to re-emerge. Understanding how protective antibody responses are generated against Zika virus can help in the development of a safe and effective vaccine. One main challenge is that Zika virus co-circulates with related viruses like dengue, such that prior exposure to one can generate cross-reactive antibodies against the other which may enhance infection and disease from the second virus. In this study, we sought to understand how prior dengue virus infection impacts subsequent immunity to Zika virus by single-cell sequencing of antibody producing cells in a second Zika virus infection. Identifying specific qualities of Zika virus immunity that are modulated by prior dengue virus immunity will enable optimal immunization strategies.

## INTRODUCTION

In 2015–2016, Zika virus (ZIKV) emerged in dengue virus (DENV) endemic regions of the Americas, causing 1.6 million infections and at least 11,000 cases of newborn microcephaly in Brazil (1, 2). ZIKV is transmitted by mosquitoes and congenitally. Infections in pregnancy lead to the greatest disease burden of neurodevelopmental delays and microcephaly in 5%–8% of Zika-exposed newborns (3). A primary concern in the development of vaccines and immunotherapies is that ZIKV co-circulates with other flaviviruses such that it is possible for most of the at-risk population to be sequentially exposed to diverse flaviviruses (4, 5).

Flaviviruses have highly conserved and immunodominant envelope (E) proteins, such that an infection can generate cross-reactive antibodies against another related virus (6–9). While some cross-reactive antibodies have no effect or even protect from subsequent disease, others can mediate antibody-dependent enhancement (ADE) of subsequent viral infection and worsen disease (10–14). ADE can occur when immune complexes of flavivirus virions and cross-reactive IgG interact with Fcγ receptors on susceptible cells, facilitating virus uptake into host cells (14–19). For example, pre-existing ZIKV immunity worsens subsequent DENV-2 and DENV-3 disease (20). Also, DENV infections are predicted to provide temporary cross-protection against ZIKV disease followed by a period of enhanced risk (21). Thus, it is important to understand how cross-reactive antibodies arise over multiple flavivirus infections.

Typically, primary infection generates flavivirus-reactive antibodies and long-lived memory B cells (MBCs) (22–25). Upon subsequent infection, pre-existing MBCs and newly stimulated B cells can rapidly differentiate into antibody-secreting cells which are called plasmablasts (PBs) (23, 25–27). Flavivirus infections, in particular, are characterized by very high frequencies of PBs during acute infection, suggesting that this cell type has a critical role in the antiviral immune response (28–30). Unlike other B cell subsets, PBs are antibody-secreting cells and thought to underlie the circulating antibodies during infection (31). In the long-term, a subset of PBs is retained in the bone marrow as long-lived antibody-secreting cells and generates the circulating antibodies that a subsequent flavivirus will encounter before new antibodies are produced by re-activated B cells (32). While PBs from flavivirus infections are found to contain both neutralizing and enhancing antibody specificities, some of the most potent flavivirus-neutralizing antibodies were derived from PBs (33–39). PBs can accumulate mutations to increase in affinity and neutralization potency against an incoming virus, so this represents an adaptable and functional subset of B cells (34, 40). Because PBs can derive from prior memory B cells, are highly frequent during the pivotal time of acute infection, and can shape the long-lasting and neutralizing antibody compartment, this cell type is one of the key drivers of immune imprinting across multiple infections. Understanding the characteristics of this compartment can explain how immune responses are modulated across multiple flavivirus infections. Indeed, ZIKV-reactive PBs from DENV-immune donors demonstrate more biased clonal repertoires and specificity to previous viruses than those with primary ZIKV infections, suggesting that flavivirus infection history impacts subsequent B cell immunity (34, 37).

In this study, we sought to understand how prior DENV serotype 3 (DENV-3) infection modulates PB responses in secondary ZIKV infection and distinguish DENV-3-primed cross-reactive signatures from ZIKV-only immunity. We conducted single-cell sequencing of PBs responding to ZIKV infection in order to define specific biases in the repertoire due to differential pre-existing DENV immunity. We hypothesized that cross-reactive antibodies across a heterologous DENV and ZIKV infection would be encoded by distinct PB immunoglobulin variable genes from that of primary or repeat ZIKV infection. We studied immune responses in ZIKV followed by DENV-3 infection because this recapitulates the sequences of flavivirus epidemics in Latin America (41, 42). We sought to model this sequence of infections with rhesus macaques to allow control of the number of prior exposures and collect blood samples at both baseline and peak PB response for each ZIKV infection. To delineate distinct contributions of prior DENV-3 on ZIKV immunity, we intensively studied the PB responses in a small number of rhesus monkeys with distinct flavivirus exposure status: primary ZIKV infection (*n* = 2), sequential ZIKV infection (*n* = 2), and a DENV-3 then ZIKV infection (*n* = 2). We performed an in-depth PB isolation, phenotyping, and Ig receptor sequencing to compare PB activation, immunoglobulin variable region gene usage, clonal diversity, somatic hypermutation, and ZIKV and DENV-3 reactivity of mAbs derived from isolated single PBs across groups. Our single-cell sequence data from PBs allowed more granular insights on immune imprinting, with the ability to track B cell clones, than cross-reactivity observed in serum antibodies. We found that ZIKV infection after prior DENV demonstrated comparable PB activation levels and formation of ZIKV-neutralizing antibodies but differential IgG isotype usage among ZIKV-reactive PBs, less diversity of clones in the PB repertoire, and lower somatic hypermutation compared with primary and secondary ZIKV-only infections in these macaques. Differences in qualities of PB responses based on infection history supports imprinting from DENV-3 onto ZIKV immunity, and this may have implications for disease outcomes of subsequent infection and vaccination in a setting of multiple flavivirus exposures.

## RESULTS

### Primary and secondary ZIKV challenge groups

**Fig 1:**
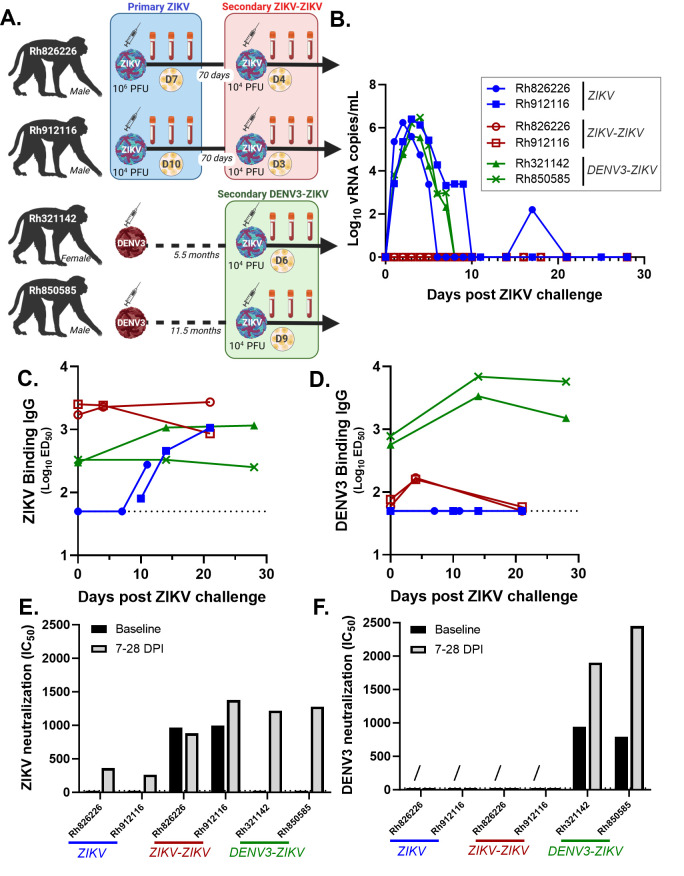
Cross-reactive antibody responses upon ZIKV infection with and without prior DENV-3 immunity. (**A**) Three distinct immunological states were generated for this study using four macaques with different sequences of ZIKV and DENV-3 challenge: (A) primary ZIKV (blue), (**B**) secondary ZIKV after prior ZIKV (red), and (C) secondary ZIKV after prior DENV-3 (green). Two macaques (Rh826226 and 912116) were first infected with ZIKV-FP, which allowed us to sample the primary ZIKV immune response (group: ZIKV). Then, these same two macaques (Rh826226 and 912116) were administered another ZIKV-FP challenge 70 days later, allowing us to sample the secondary ZIKV immune response after prior ZIKV infection (group: ZIKV-ZIKV). Two other macaques with prior DENV-3 infection (Indonesia/Sleman/1978; 6 × 10^5^ PFU) were re-challenged with ZIKV-FP 5.5 months (Rh321142) or 11.5 months later (Rh850585), allowing us to sample a secondary ZIKV immune response after prior DENV-3 infection (group: DENV-3-ZIKV). Virus inoculum dose indicated in PFU and indicated with a virion and syringe image. Plasma and PBMCs were collected up to 28 days after every ZIKV challenge and cryopreserved for use in this study (blood vial image). The yellow circle with cells indicates the timepoint at which PBs were studied in days post ZIKV challenge (“D”). (**B**) Plasma Zika viremia was measured as viral RNA copies/mL (43, 44). (**C and D**) Magnitude of ZIKV (PRVABC59) and DENV-3 (CH54389) virion binding IgG responses upon ZIKV challenge measured by virion capture ELISA. The estimated dilution at 50% of maximal binding (ED_50_) was calculated from serial dilutions of plasma and is shown over days post ZIKV challenge. Dotted line shows the average background of assay without plasma. (**E and F**) ZIKV and DENV-3 neutralization by plasma antibody before (i.e., baseline and black color) and after (7–28 days; gray color) ZIKV challenge. Plasma was serially diluted, and neutralization was assessed as 50% maximal infectivity relative to the virus alone condition. Non-neutralizing plasma is shown with a titer of 25 (first dilution). A diagonal line (/) indicates samples tested by focus reduction neutralization test only due to limiting sample availability.

We utilized cryopreserved plasma and PBMC samples from prior studies of ZIKV infections in rhesus macaques for more in-depth analysis of the early PB response here (43, 44). Two flavivirus-naïve rhesus macaques (Rh 91 and 82) were inoculated with ZIKV, constituting a primary ZIKV infection. The same two rhesus macaques were then re-challenged with ZIKV at 70 days post primary ZIKV challenge, constituting a secondary ZIKV exposure (ZIKV-ZIKV). A separate group of two rhesus macaques (Rh 32 and 85) with primary DENV-3 infection 5.5 and 11.5 months prior was inoculated with ZIKV, constituting a secondary ZIKV infection with prior DENV immunity (DENV-3-ZIKV; Fig. 1A). Plasma ZIKV viral load peaked 3 days post challenge (DPC) at 6 log_10_ viral RNA copies per mL (vRNA/mL) and was equally high in the primary ZIKV and DENV-3-ZIKV groups. Viremia resolved by 6–10 DPC, except for brief recrudescence in primary ZIKV of Rh 826226 at 17 DPC. Viremia was not detected in the ZIKV-ZIKV group (Fig. 1B). Thus, primary ZIKV infection was associated with protection from the second ZIKV infection within the 70-day interval, whereas prior DENV-3 was not associated with protection from subsequent ZIKV infection.

### Antibody cross-reactivity across ZIKV and DENV-3

We first assessed the development of plasma ZIKV-specific versus DENV-3 cross-reactive IgG responses by group. ZIKV-binding IgG were present at baseline in the secondary ZIKV-ZIKV (Mean EC_50_:2,116) and DENV-3-ZIKV (Mean EC_50_:313) groups (Fig. 1C) and DENV-3 binding IgG (ZIKV-ZIKV Mean EC_50_:66 and DENV-3-ZIKV Mean EC_50_:671) groups (Fig. 1D). As expected, the magnitude of baseline virus-binding IgG to the prior infecting flavivirus was 6–10-fold higher than to the secondary challenge flavivirus. Thus, our model recapitulates priming of flavivirus cross-reactive antibodies by primary ZIKV and DENV-3 challenge. Then, we assessed whether plasma IgG responses to ZIKV were successfully established in secondary ZIKV exposure despite prior infection with different challenge viruses. Indeed, the magnitude of ZIKV-neutralizing antibodies in the secondary ZIKV-ZIKV (Mean IC_50_:1,128) and DENV3-ZIKV (Mean IC_50_:1,247) infection groups was similarly high, suggesting formation of a productive antibody response to ZIKV (Fig. 1E).

To evaluate the role of cross-reactive DENV-3 immunity upon subsequent ZIKV infection, we measured plasma DENV-3-specific IgG responses following this infection. DENV-3 binding IgG (Fig. 1D) and neutralizing antibodies (Fig. 1F) were boosted sevenfold and twofold, respectively, within 14 days upon ZIKV challenge in the DENV-3-ZIKV group, which indicates a recall response in secondary ZIKV infection. Also, in the secondary ZIKV-ZIKV group, we detected temporary twofold boost in DENV-3 cross-reactive IgG by 4 DPC (Mean IC_50_:163) but not in the primary ZIKV challenge, supporting qualitatively distinct flavivirus antibody responses in primary versus secondary ZIKV infections (Fig. 1D). Intriguingly, throughout the ZIKV viremic period, the DENV-3-ZIKV group maintained high levels of DENV-3 binding IgG (Mean IC_50_ at 14 DPC:5,128; Fig. 1D) compared with that of ZIKV-binding IgG (Mean IC_50_ at 14 DPC:703; Fig. 1C). Together, these data support antibody-based immune imprinting from primary DENV-3 onto subsequent ZIKV immunity.

### Magnitude of activated PBs in ZIKV-ZIKV and DENV-3-ZIKV infections

**Fig 2:**
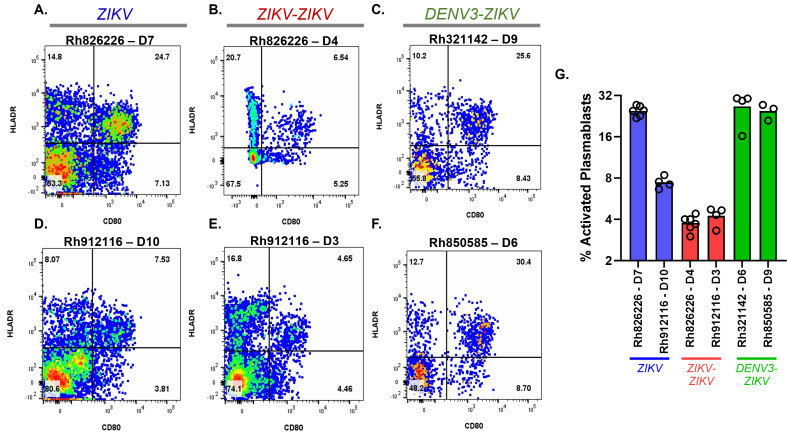
Different proportions of activated PB in primary and secondary ZIKV infection with and without prior DENV-3 immunity. (**A–F**) Activated PBs from each macaque were enumerated by flow cytometry within the first 10 days post ZIKV challenge (DPC) as viable CD14-/CD16-/CD20-/CD3-/CD123-/CD11c-/CD80+/HLADR+ (top-right quadrant). Proportion as a percentage of parent gate is indicated. (**G**) Mean percent-activated PBs for each macaque and timepoint. Circles indicate 4–6 technical replicate-stained tubes from the same PBMC sample assessed by flow cytometry on the same day. Colors indicate challenge group: primary ZIKV challenge group (blue), secondary ZIKV-ZIKV group (red), and secondary DENV-3-ZIKV group (green). Sample was collected at the indicated days post ZIKV challenge (D3–D10) based on timing of anticipated peak PB activation kinetics in primary versus secondary infections. PBMCs were cryopreserved for use in this study. See also Fig. S1.

To assess if pre-existing immunity dampens subsequent response to the same or related flavivirus on a cellular level, we compared the magnitude of activated PBs upon ZIKV challenge across groups (Fig. 2A through F). The frequency of PBs was assessed from available cryopreserved PBMCs collected at 3–10 days post ZIKV challenge since this range typically encompasses the peak PB response to infection (44). An established negative selection strategy was applied to phenotype circulating rhesus macaque PBs (Fig. S1) (45). Data are reported for each animal as means of 4–6 technical replicates from a single timepoint. As expected, the activated PB response in the ZIKV-ZIKV group (Mean: 3%–4% of B cells) was lower than that in primary ZIKV infection (Mean: 7%–24% of B cells), given that immunity was effectively established to the homologous virus in primary infection. Notably, we found that the frequency of activated PBs in DENV-3-ZIKV infection (Mean: 24%–26% of B cells) was higher than that in the ZIKV-ZIKV infection group (Mean: 3%–4% of B cells) and comparable to primary ZIKV infection (Mean: 7%–24% of B cells), suggesting that prior DENV-3 exposure was not associated with low PB responses to ZIKV infection (Fig. 2G).

### Immunoglobulin variable gene expression in ZIKV-ZIKV and DENV-3-ZIKV infections

**Fig 3:**
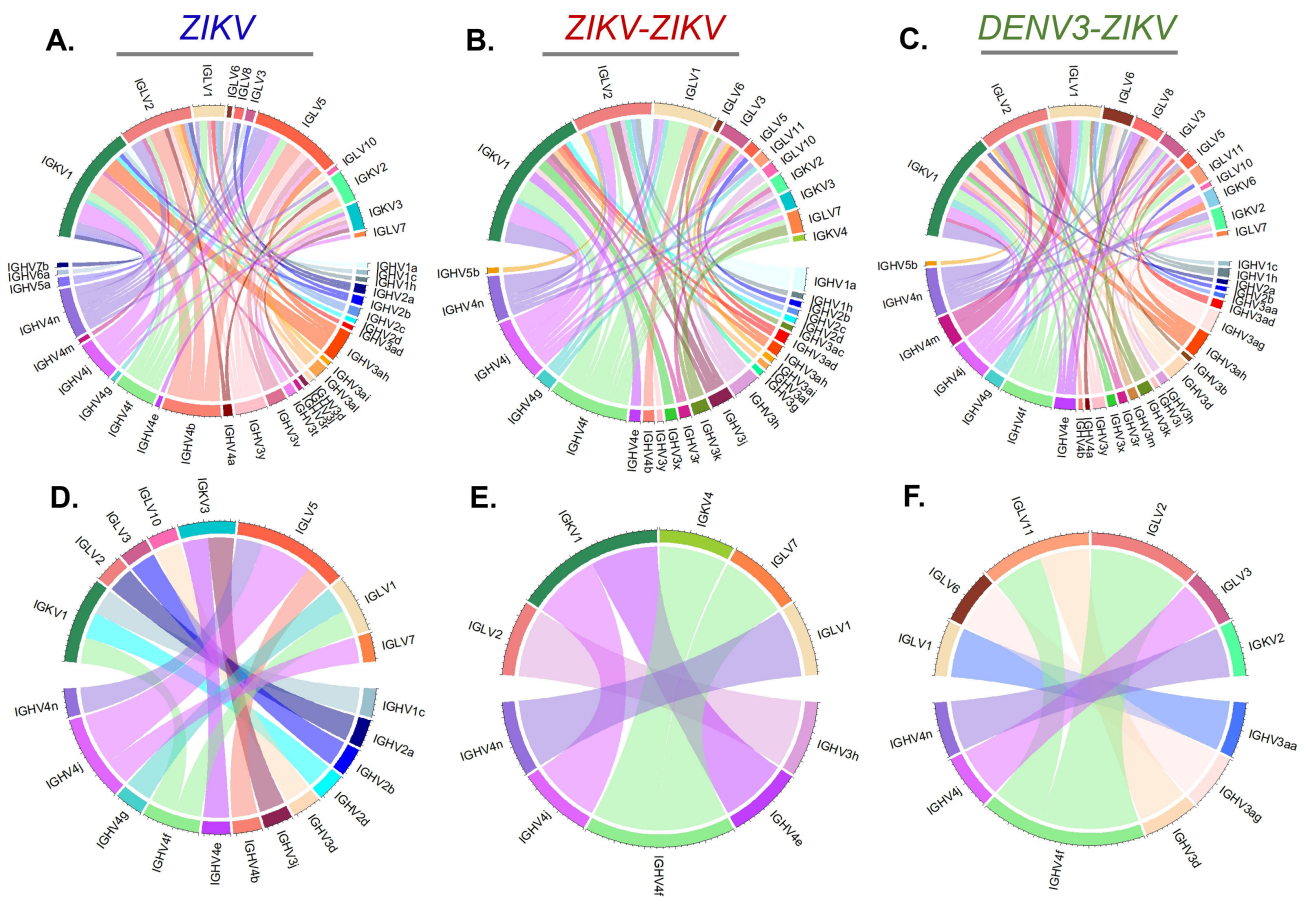
Frequencies of paired V_H_-V_L_ gene use in early activated PBs upon ZIKV challenge with or without prior DENV-3 immunity. Relative frequency of unique V_H_ gene and V_L_ gene family pairs in *all* sampled PBs per ZIKV challenge group: (**A**) primary ZIKV, (**B**) secondary ZIKV-ZIKV, and (**C**) secondary DENV-3-ZIKV groups. Paired V_H_-V_L_ from PBs were transfected into 293T cells for production of rhesus IgG1 monoclonal antibodies, which were screened for ZIKV reactivity by ELISA. ZIKV-reactivity was defined as one standard deviation above the mean reactivity of a seronegative sample at 1:1,000 dilution. V_H_ gene and V_L_ gene pairing frequencies among the subset of 29 *ZIKV-reactive* PBs by (**D**) primary ZIKV, (**E**) secondary ZIKV-ZIKV, and (**F**) secondary DENV-3-ZIKV challenge groups. Frequency shown by chord thickness. V_H_ genes are indicated in the southern hemi-circle and paired V_L_ gene families on the northern hemi-circle. Coloration is based on V_H_ gene and consistent across diagrams. Each V_H_ gene is a distinct color. See Fig. S2.

Since each B cell receptor contains a unique recombination of variable heavy (V_H_) and light (V_L_) chain genes that corresponds to antigenic specificity, we hypothesized that distinct gene usage or pairing signatures will delineate characteristics of ZIKV-only versus DENV-3-ZIKV infection cross-reactive immunity. We sequenced 363 single-sorted PBs from the expected peak PB responses (Fig. 2A through F) and assessed V_H_ and V_L_ gene usage and pairing frequencies. V_H_-[4f, 4j, and 4n] heavy chains and Vκ1, Vλ2, Vλ1 light chains were highly expanded in PBs and shared across groups, suggesting B cell selection of these receptor genes in response to ZIKV (Fig. 3A through C). Interestingly, V_H_-[3ag, 3aa, 3b, 3m] were found only in the DENV-3-ZIKV group (Fig. 3C), suggesting that these genes may represent pathways to ZIKV immunity from a DENV-immune repertoire, whereas V_H_3-j V_H_3-ai, V_H_3-al were found only expressed in ZIKV infections and not DENV-3-ZIKV, suggesting that these genes may reflect pathways to ZIKV immunity from a DENV-naïve repertoire.

We then used functional V_H_ and V_L_ pairs from the PBs sequences from the peak timepoint across primary ZIKV, ZIKV-ZIKV, and DENV-3-ZIKV groups to generate recombinant rhesus IgG1 monoclonal antibodies and screened for ZIKV-reactivity. A total of 29 out of 177 Ig-expressing supernatants from PB V_H_ and V_L_ sequences were found to be ZIKV-reactive (Fig. S2 and S3; Table 1). These ZIKV-reactive clones consisted of highly expanded genes (e.g., V_H_-4f, 4j, and 4n; Vκ1; and Vλ2), some genes unique to DENV-3-ZIKV (e.g., V_H_-3ag, 3aa), and a few low frequency genes (e.g., V_H_4g, V_H_4b, V_H_3h, V_H_3d, and Vκ1e; Fig. 3D through F; Table 1). The closest human genes to these macaque variable genes are as follows: V_H_-[4-38, 4-4, 4-59, 3-49, 3-23, 3-66], Vλ1–51, and Vκ1–5 (Table S1). Most of these have been previously identified to bind and neutralize ZIKV, DENV, and some even to the related yellow fever virus (9, 34, 46–50). Thus, we identified many common V_H_ and V_L_ gene use patterns in the early ZIKV PBs despite different prior flavivirus infection, and gene use patterns in macaques were similar to human B cell responses to flaviviruses.

**TABLE 1:** Immunogenetic features of V_H_ and V_L_ gene use and somatic hypermutation (SHM) in the subset of ZIKV-reactive rhesus PBs

Rhesus ID	DPI	IgG (ug/mL)	ZIKV binding OD_450_	Heavy chain							Light chain				
				Heavy ID	V gene	D gene	J gene	SHM (%)	CDR3 (aa)	Isotype	Light ID	V gene	J gene	SHM (%)	CDR3 length (aa)
*ZIKV*															
Rh826226	7	14.656	0.16	H010678	IGHV4-f*03	IGHD1-B*01	IGHJ5-1*01	4.12	13	A	L004136	IGLV1-f*07	IGLJ2*03	8.24	11
Rh826226	7	7.124	0.28	H010684	IGHV4-j*02	IGHD1-12*01	IGHJ5-1*01	6.60	15	A	L004142	IGLV5-b*01	IGLJ1*01	2.78	9
Rh826226	7	1.799	0.15	H010685	IGHV3-j*02	IGHD6-11*01	IGHJ4*01	3.06	13	A	K008012	IGKV3-f*06	IGKJ1-1*01	5.51	8
Rh826226	7	1.671	0.50	H010686	IGHV2-b*04	IGHD6-6*01	IGHJ5-1*01	5.84	18	M	L004144	IGLV3-l*01	IGLJ1*01	0.77	13
Rh826226	7	0.96	0.20	H010688	IGHV4-j*02	No D	IGHJ5-1*01	3.82	19	A	L004146	IGLV5-b*01	IGLJ1*01	2.78	9
Rh826226	7	2.331	0.20	H010690	IGHV3-d*02	IGHD2-8*01	IGHJ6*01	6.60	7	G	L004148	IGLV10-a*01	IGLJ3*01	3.37	11
Rh826226	7	8.455	0.17	H010691	IGHV4-j*03	IGHD1-12*01	IGHJ5-1*01	6.60	15	A	L004149	IGLV7-a*01	IGLJ7*02	35.00	0
Rh826226	7	5.751	0.16	H010692	IGHV4-n*01	IGHD5-5*01	IGHJ4*01	7.90	17	A	L004150	IGLV5-b*01	IGLJ1*01	2.78	9
Rh826226	7	5.344	0.18	H010693	IGHV4-b*02	IGHD7-A*01	IGHJ5-1*01	6.94	14	A	L004151	IGLV5-b*01	IGLJ1*01	2.78	9
Rh912116	10	5.816	0.41	H009823	IGHV4-e*01	IGHD3-21*01	IGHJ4*01	16.67	18	A	K007461	IGKV3-a*08	IGKJ1-1*01	7.20	9
Rh912116	10	0.245	2.58	H009824	IGHV1-c*01	IGHD3-26*01	IGHJ5-1*01	2.78	18	G	K007464	IGKV1-e*07	IGKJ3-1*01	0.00	9
Rh912116	10	9.732	0.26	H009828	IGHV2-d*01	IGHD1-39*02	IGHJ4*01	8.93	12	A	K007466	IGKV1-g*04	IGKJ1-1*01	8.33	9
Rh912116	10	8.186	0.40	H009830	IGHV2-a*02	IGHD2-8*01	IGHJ3*01	0.00	19	M	L003664	IGLV2-e*01	IGLJ1*01	0.00	10
Rh912116	10	9.815	1.05	H009839	IGHV4-f*02	IGHD1-39*02	IGHJ5-2*01	1.03	15	G	K007472	IGKV1-e*05	IGKJ2-1*01	0.00	9
Rh912116	10	NA	0.27	H009832	IGHV4-g*02	IGHD5-5*02	IGHJ4*01	12.85	11	A	L003666	IGLV1-i*02	IGLJ5*01	8.61	0
*ZIKV-ZIKV*															
Rh826226	4	6.003	1.05	H010490	IGHV3-h*02	IGHD1-1*01	IGHJ4*01	1.36	14	A	L004029	IGLV2-f*01	IGLJ1*01	1.48	10
Rh826226	4	NA	0.36	H010502	IGHV4-e*01	IGHD3-9*01	IGHJ6*01	7.29	10	M	K007881	IGKV1-e*07	IGKJ3-1*01	1.52	9
Rh826226	4	2.139	0.22	H010509	IGHV4-f*03	IGHD3-9*01	IGHJ4*01	0.00	13	A	K007884	IGKV4-a*03	IGKJ4-1*01	0.35	9
Rh826226	4	2.242	0.19	H010524	IGHV4-j*02	IGHD4-4*01	IGHJ4*01	7.22	12	A	K007895	IGKV1-g*03	IGKJ1-1*01	1.52	9
Rh912116	3	6.4	0.80	H691397	IGHV4-n*03	IGHD2-35*01	IGHJ1*01	17.53	17	A	L691029	IGLV1-e*01	IGLJ1*01	8.99	11
Rh912116	3	2.4	0.38	H691426	IGHV4-f*03	IGHD4-27*01	IGHJ4*01	11.00	8	A	L691054	IGLV7-c*01	IGLJ3*01	4.81	9
*DENV3-ZIKV*															
Rh850585	6	1.55	1.96	H691441	IGHV3-ag*01	IGHD1-12*01	IGHJ4*01	9.03	15	G	L691068	IGLV6-c*01	IGLJ2*03	2.56	10
Rh321142	9	8.55	0.41	H691010	IGHV4-f*02	IGHD5-23*01	IGHJ5-1*01	7.56	12	M	L690735	IGLV11-a*01	IGLJ6*01	2.08	10
Rh321142	9	> 13.85	1.40	H691018	IGHV4-j*02	IGHD5-5*01	IGHJ5-1*01	4.51	10	G	L690741	IGLV3-j*05	IGLJ2*02	6.13	11
Rh321142	9	5.95	0.34	H691029	IGHV4-f*03	IGHD2-20*02	IGHJ5-1*01	5.15	19	G	L690749	IGLV2-j*15	IGLJ2*02	0.00	10
Rh321142	9	5.75	0.88	H691030	IGHV3-aa*02	IGHD5-5*01	IGHJ4*01	6.67	14	G	L690750	IGLV1-e*01	IGLJ1*01	2.62	11
Rh321142	9	4.85	0.44	H691031	IGHV3-d*02	IGHD1-7*01	IGHJ4*01	2.78	15	G	L690751	IGLV11-a*01	IGLJ3*01	0.69	9
Rh321142	9	3.85	0.37	H691038	IGHV4-f*03	IGHD6-24*01	IGHJ1*01	6.53	13	A	L690756	IGLV2-c*02	IGLJ1*01	1.48	10
Rh321142	9	1.65	0.92	H691053	IGHV4-n*03	IGHD3-9*01	IGHJ5-2*01	6.19	21	G	K690319	IGKV2-o*01	IGKJ2-1*01	1.43	9

### Diversity of PB gene use following ZIKV infection and reinfection

Next, we evaluated for immune focusing upon secondary ZIKV infection through differences in Shannon Diversity Index (H) of PB V_H_ genes and V_L_ gene families across groups. Among all 363 PBs, no significant difference was observed in Shannon’s H for V_H_ and V_L_ diversity across macaques (Fig. S4A and B). However, in the subset of 29 ZIKV-reactive PBs, diversity of V_H_ genes was significantly higher in primary ZIKV infection than secondary ZIKV-ZIKV or DENV3-ZIKV infection, suggesting immune focusing in secondary ZIKV-reactive PBs despite primary infection with different flaviviruses (*P* < 0.05, Hutcheson *t*-test; Fig. S4C and D). Moreover, there were no significant differences in variable gene diversity across ZIKV-ZIKV and DENV3-ZIKV groups. Together, this indicates that the PB clonal diversity of a ZIKV infection after prior DENV3 is unlike a primary ZIKV infection and has similarly low diversity as multiple ZIKV exposures.

### Distribution of PB isotypes following ZIKV-ZIKV and ZIKV-DENV-3 infection

**Fig 4:**
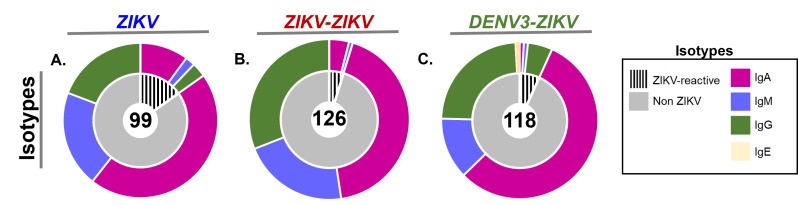
PB immunoglobulin isotype distribution by ZIKV reactivity and challenge group. Immunoglobulin rhesus isotype distribution inferred by PB sequence and shown by (**A**) ZIKV, (**B**) ZIKV-ZIKV, and (**C**) DENV-3-ZIKV challenge groups. Colors in outer pie indicate isotype: IgA (magenta), IgM (purple), IgG (green), and IgE (yellow). Inner pie chart shows ZIKV-reactive PBs in the pie slice with black dashed lines and non-reactive PBs in gray pie slices.

Since antibody isotype also affects function and cross-reactivity, we assessed the Ig isotype distribution from 363 V_H_ constant region sequences in total PBs and 29 V_H_ in ZIKV-reactive PBs. While IgG is traditionally considered the most functional isotype against viruses and is most abundant in plasma, surprisingly, IgA predominated the early PB compartment (Fig. 4). There were significantly more IgA-expressing ZIKV-reactive PBs in the ZIKV and ZIKV-ZIKV infection groups, as compared with the DENV3-ZIKV infection group (*P* < 0.05, Fisher’s exact test). In contrast, there were significantly more IgG-expressing ZIKV-reactive PBs in the DENV3-ZIKV animals as compared with the ZIKV and ZIKV-ZIKV infection groups (*P* < 0.05, Fisher’s exact test). Yet, IgA abundance in ZIKV-reactive PBs in the ZIKV-ZIKV group did not correspond to increases in the total plasma IgA (Fig. S5). Also, there were significantly more IgM-expressing PBs in the ZIKV-ZIKV infection group than the DENV3-ZIKV infection group (*P* < 0.05, Fishers exact test; Fig. 4B and C). As expected, IgG-expressing PBs were more frequent than IgM-expressing PBs in secondary ZIKV-ZIKV as compared with primary ZIKV infection (Fig. 4A and B). Thus, IgA-expressing PBs are a common feature across groups but were more frequently represented in ZIKV-reactive clones in ZIKV-only infections [66% (*n* = 10/15) and 83%(*n* = 5/6)] than with prior DENV-3 immunity (12.5%, *n* = 1/8).

### Differential somatic hypermutation in PBs following ZIKV-ZIKV and DENV-3-ZIKV infection

**Fig 5:**
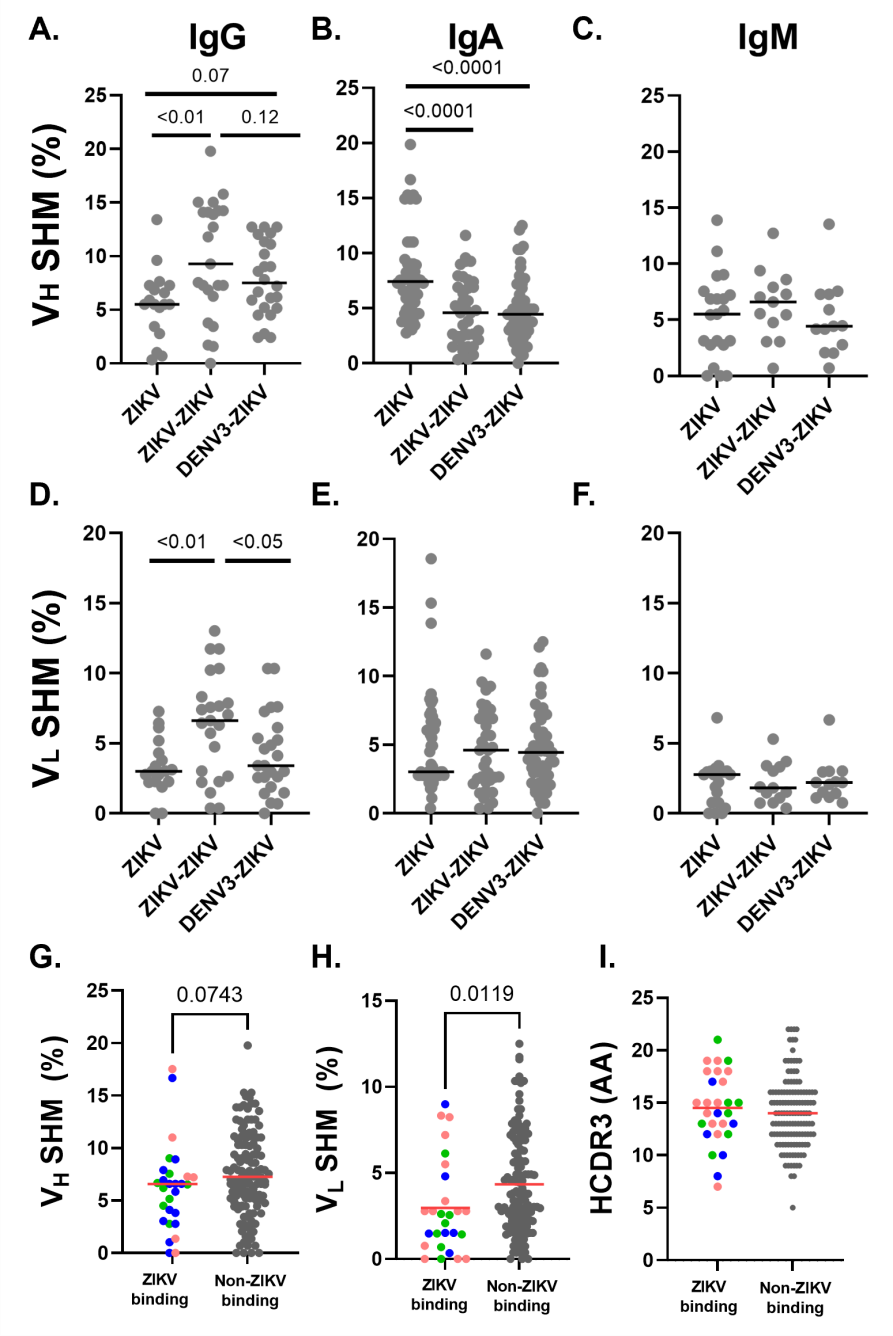
Differences in somatic hypermutation in PBs upon ZIKV challenge based on prior flavivirus immunity and ZIKV-reactivity. The V_H_ and V_L_ sequences of PBs were assessed for percent SHM relative to a reference Rhesus immunoglobulin sequence (Clonalyst). Amino acid length of the V_H_ complementarity determining region (HCDR3) was inferred from V_H_ sequence (Clonalyst). Percent SHM of the V_H_ gene was assessed in PBs from each primary ZIKV, secondary ZIKV-ZIKV, and secondary DENV-3-ZIKV challenge groups by IgG (**A**), IgA (**B**), and IgM (**C**) isotypes. Percent SHM of the V_L_ gene was assessed in PBs from each ZIKV challenge group by IgG (**D**), IgA (**E**), and IgM (**F**) isotypes. Comparison of V_H_ SHM (**G**) and V_L_ SHM (**H**) in PBs that produced Zika virion-reactive antibodies to those that were not ZIKV-reactive. (I) Length of HCDR3 of PBs by ZIKV-reactivity. Each dot represents a single functional sequence from PBs. Line at median indicated on all plots. Groups were compared for significant differences by Mann-Whitney test without multiple corrections. *P* value estimates of significantly different groups are indicated on the graphs. See Fig. S5.

B cell responses mature in affinity through SHM in the V_H_ and V_L_ regions over repeat exposure to the same antigen. We sought to understand the extent of SHM following primary ZIKV and secondary ZIKV-ZIKV infections and whether prior exposure to DENV-3 differentially impacts SHM in secondary ZIKV infection. We found that V_H_ and V_L_ gene SHM was significantly higher in IgG-expressing PBs following ZIKV-ZIKV infection than primary ZIKV infection, suggesting that SHM occurred in both V_H_ and V_L_ over time or with repeat ZIKV exposure (*P* < 0.05, Mann-Whitney test; Fig. 5A). Also, IgG V_L_ gene SHM was significantly higher following ZIKV-ZIKV re-challenge than DENV-3-ZIKV infection (*P* < 0.05), and IgG V_H_ was trending higher as well (*P* = 0.12, Mann Whiteny tests; Fig. 5D). Thus, homologous secondary ZIKV-ZIKV infection results in higher SHM in IgG-expressing PBs than heterologous DENV-3-ZIKV infection, even though IgG PBs were more abundant in DENV3-ZIKV infection (Fig. 4C). Unlike IgG PBs, IgA-expressing PBs did not demonstrate significant differences between the ZIKV-ZIKV and DENV-3-ZIKV infection groups (Fig. 5B and E). However, IgA V_H_ SHM was significantly higher in primary ZIKV as compared with either secondary ZIKV-ZIKV or DENV-3-ZIKV infection (*P* < 0.05, Mann-Whitney test; Fig. 5B). As expected, IgM V_H_ and V_L_ SHM were not significantly different across groups (Fig. 5C and F). Thus, high IgA V_H_ SHM in PBs was characteristic of primary ZIKV infection, whereas high V_H_ mutations in IgG-expressing PBs distinguish repeat ZIKV-ZIKV infections from that of ZIKV infection with prior DENV-3 immunity.

Modest levels of SHM may suffice to generate ZIKV-reactivity, since these IgG have lower V_H_ SHM and significantly lower V_L_ SHM compared with non-ZIKV-reactive clones (*P* < 0.05, Mann-Whitney test, Fig. 5G and H). Interestingly, IgA isotype ZIKV-reactive PBs demonstrated higher median V_H_ SHM (6.9%) as compared with non-IgA-expressing ZIKV-reactive PBs (5.4%; *P* < 0.05, Mann-Whitney test; Table 1). Also, the V_H_ complementarity determining region 3 (HCDR3) amino acid length, the hypervariable region that accumulates antigen-binding mutations, was not significantly different between ZIKV-reactive and non-reactive PBs or across groups (Fig. 5I). In sum, ZIKV-reactive PBs were characterized by median V_H_ SHM of 6.6%, median V_L_ SHM of 2.6%, and median HCDR3 of 15 residues, with IgA-expressing ZIKV-specific PBs representing the most mutated specificities.

### Flavivirus neutralization by PB-derived monoclonal antibodies

**Fig 6:**
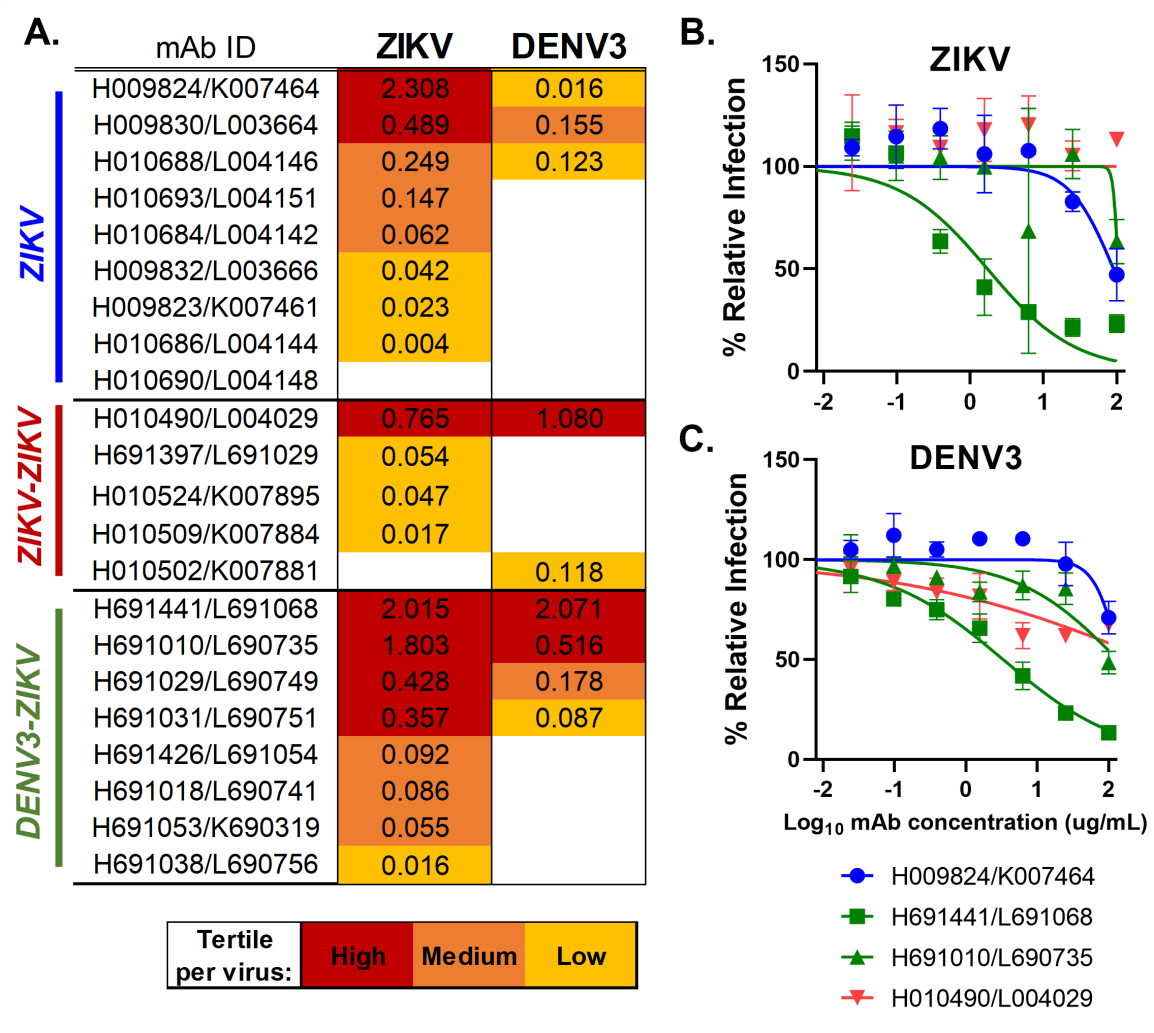
Assessment of recombinantly generated mAbs for ZIKV and DENV-3 binding and neutralization. Recombinant IgG mAbs were produced from the sequence of 22 highest ZIKV-reactive PBs across macaques. (A) Heat map of the magnitude of ZIKV (strain PRVABC59) and DENV-3 (strain CH54389) whole virion binding at 25 µg/mL of IgG mAb shown as mean optical density at 450 nm (OD_450_) of two replicates by challenge groups: primary ZIKV (blue), secondary ZIKV-ZIKV (red), and secondary DENV-3-ZIKV (green). All positive values colored by tertile to demonstrate the strength of binding as high (magenta), medium (orange), and low (yellow). Four mAbs representing the top 10% of ZIKV-binders were assessed for ZIKV (**B**) and DENV-3 (**C**) neutralization. Percent infection of ZIKV (strain PRVABC59) and DENV-3 (strain CH54389) over a serial dilution of mAb was calculated relative to virus only control and fit to a sigmoidal curve. Each mAb was tested with two replicates. Coloration by challenge group of original PBs.

Of the 177 Ig-expressing supernatants isolated from PBs of all macaques, 22 were in the top quartile of ZIKV-reactive intensities and were produced recombinantly as Rhesus IgG mAb to assess ZIKV and DENV-3 neutralization (Fig. S3). Of the 22 recombinantly produced IgG mAbs, 20 were confirmed to bind to ZIKV and 9 to DENV-3 (Fig. 6A). The DENV-3-ZIKV infection group also generated 50% (4/8) high ZIKV-binding IgG clones, of which half (2/4) also demonstrated high cross-reactivity with DENV-3 (Fig. 6A). The other 50% (4/8) of high ZIKV-binding clones isolated from the DENV-3-ZIKV infection group demonstrated medium-to-low ZIKV-reactivity and were not cross-reactive to DENV-3 (Fig. 6A). Subsequently, the highest ZIKV-binding IgG mAbs (*n* = 4) were further tested for cross-neutralization, revealing that mAb H691441/L691068 isolated from the DENV-3-ZIKV infection group neutralized both ZIKV (FRNT_50_ = 1.7 µg/mL) and DENV-3 (FRNT_50_ = 3.3 µg/mL; Fig. S3). This mAb, isolated from an IgG-expressing PB in Rh850585, contains 9% V_H_ SHM and 2.5% V_L_ SHM (Table 1). Variable genes V_H_3-ag and Vλ6 were utilized in this mAb, which was not common among ZIKV-reactive clones in ZIKV infection groups without prior DENV-3 immunity (Table 1). However, the closest human gene, V_H_3-66, arose in other studies among ZIKV, DENV, and yellow fever virus-reactive mAbs (9, 46, 47). Thus, primary ZIKV and secondary ZIKV-ZIKV infection generated high ZIKV-binding IgG clones, and importantly, prior DENV-3 did not abrogate ZIKV-specific neutralizing clones during secondary infection.

## DISCUSSION

In this study, we examined the contribution of pre-existing DENV-3 immunity on early ZIKV B cell immunity by conducting a detailed single-cell genomic analysis of the immunoglobulin variable regions of PBs after serial flavivirus infections. PBs are transiently expanded upon ZIKV and DENV infections and thought to be essential to the formation of protective antiviral immunity (11, 29, 51–53). Using an Indian rhesus macaque model of flavivirus infections, we generated optimal comparator groups of primary ZIKV and multiple ZIKV challenges that allowed us to specifically detect differences in secondary ZIKV PB responses that may be related to prior DENV-3. While prior studies from our group and others characterized PB response kinetics with controlled series of infections (26, 44, 54–56), here, we additionally sequenced the B cell receptor variable regions of 363 PBs and characterized 177 derivative monoclonal antibody supernatants from acute ZIKV infection to dissect the signature associated with DENV-3 imprinting onto subsequent PB immunity to ZIKV. We defined the primary B cell variable genes used, as well as differences in PB gene diversity, mutational burden, and isotypes that varied in the acute response to ZIKV in relation to prior infection.

B cell receptor variable gene recombination underlies antigen specificity and subsequent clonal selection into the antiviral immune response (31). Among ZIKV-reactive PB sequences, we identified several variable genes with human homologs constituting established ZIKV- and flavivirus-reactive specificities. Remarkably, we identified ZIKV-reactive PBs using V_H_3-d and Vκ1-e genes. These correspond to the commonly used human V_H_3-23 and Vκ1–5 genes and are likely due to convergent gene evolution within a ZIKV-specific B cell response (34, 57). These sequences may define a common mode of pan-flavivirus recognition, since derived mAbs interact with ZIKV EDIII, Zika whole virion, DENV E protein, and YFV E protein (9, 34, 46–50, 57). Also, repeated isolations of both V_H_3-23 and Vκ1–5 flavivirus-specific mAbs suggest that both of these heavy and light chains are being preferentially selected within a ZIKV immune response. Many of our other sequences matched previously reported human variable genes represented in flavivirus antibodies, including V_H_3-48, V_H_3-66, V_H_4-39, V_H_4-59, Vλ5–39, Vκ1–12, Vκ2–28, Vκ3–20, Vλ3–21, and Vλ1–51 (9, 46–50). This homology demonstrates that our macaque model recapitulated human flavivirus immunoglobulin gene use. Importantly, recurrent isolations of common light chains in the response to ZIKV infections in our data, together with prior studies, reinforce a role for B cell receptor light chains in flavivirus recognition, even though heavy chains are most often studied (34, 47, 49, 50, 57).

Although we had hypothesized that flavivirus cross-reactivity with and without prior DENV-3 would manifest in distinct PB variable gene signatures in the DENV-3 immune versus ZIKV-only infection groups, we found no clear bias in ZIKV-induced PB variable gene usage. This and prior studies reveal that both ZIKV and DENV infections are associated with some common PB genes within families V_H_3 and V_H_4 (9, 46, 48, 49). A few ZIKV-reactive PB genes may be associated with immune history, such as V_H_3-aa and V_H_3-ag (human gene: V_H_3-13 and V_H_3-66), which was found only in DENV-immune ZIKV responses, whereas V_H_3-j (human gene: V_H_3-49) was found only in ZIKV-only responses. While it remains possible that biases in gene use exist by exact flavivirus infection history, we were not able to test this with only 363 singly sequenced cells from our data set due to the enormous diversity of variable gene usage we uncovered among the acute ZIKV infection PB response, although, interestingly, V_H_3-49 was found to be DENV and yellow fever virus binding and V_H_3-66 neutralized both ZIKV and DENV-3 in this study (47, 48). The broad cross-reactivity associated with these genes cumulatively across studies suggests that these Ig genes may be recruited for antibodies that target highly conserved epitope studies (47, 48). Thus, different immune histories may provide a host with different evolutionary starting points to generate a flavivirus cross-reactive B cell repertoire.

The diversity of variable genes in the transient PB population is related to B cell selection, where low diversity indicates few distinct B cell clones present in a population and suggests preferential selection for these clones (58). Among the ZIKV-reactive PBs, we identified the highest V_H_ and V_L_ diversity levels upon primary ZIKV infection and lower levels in both homologous and heterologous secondary ZIKV responses. This transition from primary to secondary ZIKV-ZIKV is expected as anti-ZIKV B cells established in the primary infection can be preferentially selected into the secondary immune response, expand to occupy more clonal space, and thereby decrease diversity in PBs. A similar pattern of decreased diversity among circulating PBs was noted in secondary human ZIKV and DENV infections (34, 46, 57). We also hypothesized that a primary DENV-3 infection would be associated with fewer or weaker ZIKV-reactive B cells than a primary ZIKV infection, and so, there may be fewer clones to select into a secondary DENV-3-ZIKV PB response than in ZIKV-ZIKV infection. In contrast, we found that the diversity among ZIKV-reactive PBs in the DENV-3-ZIKV group was on par with ZIKV-ZIKV and significantly lower than primary ZIKV. This interesting pattern is compatible with immune imprinting and suggests that a recall response from primary DENV-3 infection may contribute to the secondary ZIKV PB response. A similar pattern has also been reported in five humans with acute ZIKV infection and pre-existing DENV immunity (34, 40). Moreover, boosting of plasma DENV-3 binding and neutralizing antibodies upon ZIKV infection in these macaques supports elicitation of a recall response in these two animals. It is possible that DENV-3 recall supports ZIKV-neutralizing antibodies since the strongest ZIKV-binding mAbs from the DENV-3-ZIKV group also cross-reacted with DENV-3, suggesting a recall origin for these specificities. Whereas mAbs from the DENV-3-ZIKV infection group that did not cross-react with DENV-3 showed lower levels of ZIKV binding, and these may be elicited *de novo*. Epidemiologically, prior DENV immunity is associated with milder subsequent ZIKV (59, 60), and our work suggests this may, in part, be due to immune focusing toward highly ZIKV-binding and neutralizing antibody specificities.

Somatic mutations in B cell variable regions can further increase the affinity and potency of the antibody response (31). We observed moderate to high levels of somatic hypermutation in the immunoglobulin heavy and light variable regions of ZIKV-reactive PBs that mirrored prior estimates from ZIKV-infected patients (34, 40, 61, 62). Due to conflicting evidence on prior DENV infections, it is unclear how SHM of PBs evaluated in our study compare with that of DENV infection (40, 48, 63). One intriguing observation is that boosting of SHM in secondary ZIKV-ZIKV infection occurred upon challenge but in the absence of measurable viremia, supporting the potential for asymptomatic/subclinical infections in flavivirus co-endemic areas to generate more mature B cells over time (64). Interestingly, ZIKV-ZIKV infection-induced PBs acquired significantly higher SHM than DENV-3-ZIKV infection-induced PBs. Thus, the identical ZIKV rechallenge is associated with more B cell maturation within a limited clonal diversity than DENV-3-ZIKV infection. It is possible that a ZIKV-ZIKV rechallenge may be better at promoting immune focusing than DENV-3-ZIKV because it is an identical repeat exposure. This phenomenon of immune focusing upon identical re-exposure to antigens was also observed with the SARS-CoV-2 mRNA vaccine booster series (65). Time since last infection can also modulate the extent of boosting in SHM. Recent studies indicate that virus-reactive germinal centers can persist up to 3–5 months, suggesting that a later infection, beyond the timeframe of our study, may also result in a bigger boost than earlier infections (66, 67). Thus, the timing between viral challenges and doses used in our study may limit our inferences. It is possible that a challenge that is administered at a later timepoint may differentially modulate the PB response. Further studies should continue to delineate the contributions of sequential related infections on B cell diversity and mutations.

Finally, the distribution of antiviral immunoglobulin isotypes impacts antibody functional profile and immune protection (12). For example, pre-existing antiviral antibodies of the IgG1, IgG4, IgA2, and IgM isotypes are correlated with protection against subsequent symptomatic DENV-3 infection (12). In our ZIKV-only challenge groups, ZIKV-reactive specificities arose from IgM and IgA PBs, whereas prior DENV-3 infection was associated with a different profile such that ZIKV-reactive specificities arose from the IgG compartment. IgG and early IgM antibodies are known to potently neutralize the virus (49, 57, 68, 69), whereas cross-reactive IgG can also enhance viral replication (49, 70–72). Intriguingly, we found a large rise of IgA+ PBs in all primary and secondary ZIKV infections, which has also been observed for DENV and SARS-CoV-2 infections, and influenza vaccination (32, 67, 73, 74). While Waickman et al. reported higher antiviral IgA-expressing PBs in primary rather than secondary DENV infection, we find similarly high levels of IgA-expressing PBs in primary and secondary ZIKV infection (73). Anti-DENV IgA were found to be effective disruptors of IgG-mediated DENV enhancement *in vitro* and were broadly neutralizing (71, 73, 75). While there is evidence on the value of IgA antibodies in DENV infections, the role for IgA in ZIKV infection remains unclear.

Small sample sizes, the number of macaques, and cryopreservation of fragile PBs were limitations of this study. Given two animals per group, the role of inter-individual variability and timing of past exposure could not be deconvoluted from infection history, our primary analytic variable. Thus, our inferences are based on associations and delineating causal relationships will require further investigation.

In summary, we found ZIKV infection after DENV-3 infection was associated with early formation of high-magnitude ZIKV-binding and neutralizing antibodies and PB responses, despite evidence of immune imprinting by recall responses at both the antibody and B cell levels. Indeed, DENV-3-ZIKV and ZIKV-ZIKV infection-induced PBs demonstrate the use of similar immunoglobulin variable genes, indicating common approaches to ZIKV recognition despite differences in prior infection. Also, acute PB responses in both secondary DENV-3-ZIKV and ZIKV-ZIKV infection are characterized by significantly lower clonal diversity than primary ZIKV infection, whereas multiple ZIKV exposures are associated with more B cell maturation via SHM. Interestingly, a higher proportion of IgA isotype PBs was found in ZIKV-only immune histories, where ZIKV-reactive IgA PBs were more somatically mutated than non-IgA ZIKV-reactive PBs. It is possible that IgA antibodies and B cells may differentially modulate anti-ZIKV immune functions based on prior exposure. Overall, immune imprinting by DENV was associated with a productive ZIKV immune response and was characterized by many shared B cell receptor genes but qualitatively distinct mutational and isotype distribution in the acute B cell response.

## MATERIALS AND METHODS

### Study design

We utilized plasma and PBMC samples from previously studied Indian-origin rhesus macaques with ZIKV and DENV-3 infections (43, 44). The primary ZIKV group (*n* = 2) comprised of 4-year-old male macaques (*Macaca mulatta*) Rh826226 and Rh912116 and was inoculated subcutaneously (SC) with 1 × 10^6^ and 1 × 10^4^ PFU/mL, respectively, of Asian-lineage ZIKV strain Zika virus/H.sapiens-tc/FRA/2013/FrenchPolynesia-01-v1c1 (ZIKV-FP; GenBank: KJ776791). For the ZIKV-ZIKV group, the same two macaques were re-challenged SC after 70 days with 1 × 10^4^ PFU ZIKV-FP. For the DENV-3-ZIKV group, two macaques were inoculated SC with 1 × 10^4^ PFU ZIKV-FP after 1 year (Rh850585, male) or 0.4 years (Rh321142, female) of 6 × 10^5^ PFU DENV-3 (dengue virus/H.sapiens-tc/IDN/1978/Sleman/78) challenge. Blood was collected at baseline and 1–14 and 21–28 DPC. PBMCs were cryopreserved upon collection and thawed immediately before experiments in this study.

### Virus production for *in vitro* assays

We used previously described methods (49). Briefly, DENV-3 was grown in C6/36 cells cultured with RPMI 1640, L-glutamine, 25 mM HEPES, 1× penicillin-streptomycin (PS), and 2% heat-inactivated fetal bovine plasma (FBS); ZIKV was grown in Vero-81 cells cultured in DMEM, with 1× MEM NEAA, 1× PS, and 10% FBS. DENV-3 strain CH54389 (GenBank: DQ863638.1) was provided by Dr. Aravinda de Silva (University of North Carolina at Chapel Hill), and ZIKV strain PRVABC59 (ZIKV-PR; GenBank: KU501215.1) was obtained from BEI.

### Viral RNA quantification from plasma

Viral RNA was isolated from plasma using the Maxwell Viral Total Nucleic Acid Purification kit on the Maxwell 48 RSC instrument (Promega). Viral RNA was then quantified using a highly sensitive Reverse transcription quantitative polymerase chain reaction (RT-qPCR) assay based on the one developed by Lanciotti et al. (2008), though the primers were modified to accommodate both Asian- and African-lineage Zika viruses. RNA was reverse transcribed and amplified using the TaqMan Fast Virus 1-Step Master Mix RT-qPCR kit (LifeTechnologies) on the LightCycler 480 (Roche) and quantified by interpolation onto a standard curve made up of serial 10-fold dilutions of *in vitro*-transcribed RNA. RNA for this standard curve was transcribed from a plasmid containing an 800-bp region of the Zika virus genome that is targeted by the RT-qPCR assay. The final reaction mixtures contained 150 ng random primers (Promega), 600 nM each primer, and 100 nM probe. Primer and probe sequences are as follows: forward primer: 5′-CGYTGCCCAACACAAGG-3′, reverse primer: 5′-CCACYAAYGTTCTTTTGCABACAT-3′ and probe: 5′−6-carboxyfluorescein-AGCCTACCTTGAYAAGCARTCAGACACYCAA-BHQ1-3′. The reactions cycled with the following conditions: 50°C for 5 minutes, 95°C for 20 seconds followed by 50 cycles of 95°C for 15 seconds, and 60°C for 1 min. The limit of detection of this assay is 150 copies/mL.

### Neutralization

We used previously described methods for the 96-well focus-forming neutralization test (FRNT) and 12-well plaque-forming neutralization test (PRNT) (44, 76). For the FRNT, virus and serial dilutions of mAb or plasma were co-incubated for an hour and then transferred to a confluent Vero cell monolayer for an hour at 37°C. An overlay of 1% methylcellulose (125 µL/well) was added for foci development. Plates with ZIKV were incubated for 40–42 hours, and plates with DENV-3 were incubated for 48 hours at 37°C. Cells were fixed with 2% paraformaldehyde for 30 minutes, and then, foci were stained with 0.5 µg/mL 4G2 and detected with anti-mouse IgG HRP (1:5,000) and TrueBlue. For the PRNT, plasma serial dilutions were combined with a set amount of virus for 1-hour co-incubation, inoculum was removed, and an overlay of 1.2% oxoid agar was applied. Cells were incubated at 37°C for 4 days for plaque development and stained with 0.33% neutral red. Foci were counted using ImmunoSpot plate reader (Cellular Technology Limited), and plaques were counted manually. The optimal mean foci count in virus-only control across FRNT plates for DENV-3 was 35–52, and that for ZIKV was 57–62. The dilution of 50% maximal infectivity relative to the virus-only control (NT_50_) was calculated with the sigmoidal dose-response (variable slope) curve in Prism 9.2.0 (GraphPad), constraining values between 0% and 100% relative infection. Percent relative infection curves and the resulting NT_50_ were considered upon quality control: if *R*^2^ > 0.65, absolute value of hill slope > 0.5, and the curve crossed 50% relative infection. Samples or mAbs that did not meet this criterion were considered non-neutralizing. Samples were assessed in replicates. Negative controls are as follows: media and HIV mAb CH22. Positive controls are as follows: rhesus hyperimmunoglobulin from ZIKV-infected macaques and DV78/DV10 mAbs (Absolute Antibody).

### Whole virion-binding ELISA

Virion capture ELISA methods were previously described (52). Briefly, high-binding 96-well plates (Greiner Bio One) were coated with 40 ng/well of 4G2 mAb in 0.1 M carbonate buffer (pH 9.6) overnight at 4°C. Plates were blocked in Tris-buffered saline containing 0.05% Tween-20 with 5% normal goat plasma for 1 hour at 37°C, followed by an incubation with either ZIKV (PRVABC59) or DENV-3 (CH54389) infectious supernatant for 1 hour at 37°C. Then, plasma or mAb samples were added in replicate and incubated for 1 hour at 37°C. Eight-point serial dilutions for plasma started at 1:12.5 with fourfold serial dilutions, and mAbs started at 100 µg/mL fourfold serial dilutions. Binding was detected with anti-macaque IgG HRP antibody (1:5,000 for 30 minutes Southern Biotech) and 100 µL/well SureBlue (KPL). Reactions were stopped by 100 µL/well of Stop Solution (KPL) after 5 minutes, and OD was detected at 450 nm (PerkinElmer). Small-scale transfection supernatants were assessed undiluted in replicate by the same approach, except for the use of goat-anti macaque IgG HRP (Abcam) at 1:2,500 dilution. Virion binding was evaluated as the dilution/concentration demonstrating 50% maximal optical density over a serial dilution, with a sigmoidal dose-response (variable slope) curve in Prism 9.2 (GraphPad) using least squares fit. This value was considered valid if the OD_450_ at 25 μg/mL was fivefold higher than block alone. Negative controls are as follows: HIV mAb Rh CH22, seronegative sera, and block alone. Positive control was rhesus hyperimmunoglobulin from ZIKV-infected macaques.

### Flow sorting of rhesus monkey plasmablasts

We sorted PBs by gating on CD14−/CD16−/CD20−/CD3−/CD123−/CD11c−/CD80+/HLA-DR+ from live leukocytes and singlets, as previously described (44, 45). Gating limits were determined by FMO controls for CD80, CD123, and CD11c. Briefly, PBMCs were stained with CD20 FITC (L27), CD80 PE (L307.4), CD123 PE-Cy7 (7G3), CD3 APC-Cy7 (SP34-2; all from BD Biosciences), CD14 AF700 (M5E2), CD11c BV421 (3.9), CD16 BV570 (3G8; all from BioLegend), and HLA-DR PE-TxRed (TÜ36; Invitrogen). A LIVE/DEAD Fixable Aqua Dead Cell Stain kit (Invitrogen) was used for viability. Cells were resuspended with PBS/1%BSA, stained with fluorescent antibodies for 30 min at 4°C, washed with 1× PBS, stained for 30 min with the LIVE/DEAD Aqua at 4°C, washed with 1× PBS and PBS/1%BSA, and then fixed in 2% paraformaldehyde. Stained PBMCs were acquired on a LSRII Flow Analyser (BD Biosciences). Single color-stained Bangs beads were used to compensate and calibrate the machine prior to acquisition. PBs were sorted on low speed into 96-well plates containing an RNA-stabilizing mixture. Plates were frozen in ethanol and dry ice and stored at −80C until reverse transcription. Cellular phenotypes were analyzed using FlowJo (Version 10.8.1).

### Nested immunoglobulin variable gene amplification

V_H_ and V_L_ genes from each well were reverse transcribed using Superscript III (Thermo Fisher), and then, cDNA was amplified under various conditions of sequential external primer sets and internal primer sets (4 µm of forward and reverse primers), as previously described (77–79). These rhesus-specific primer sets target different V_H_ gene families and V_H_ constant regions, to achieve a successful amplification of the heavy and light chain from each well (79). To assess if V_H_ regions were successfully amplified, we checked the size of PCR products on a 96-well agarose gel. Then, corresponding V_L_ were amplified with primer sets targeting kappa or lambda segments and PCR product by gel. Successfully amplified V_H_ and V_L_ were then sequenced (Genewiz). Contigs for each of the forward and reverse immunoglobulin sequences were assembled using an in-house bioinformatics pipeline for antibody sequence analysis which included aligning the constant region sequences to a library of rhesus constant regions. Variable gene usage was determined using the heavy and light chain reference gene library in Cloanalyst (https://www.bu.edu/computationalimmunology/research/software/), which also annotated percent somatic hypermutation and HCDR3 length.

### Expression of V_H_ and V_L_ from plasmablasts as IgG1 mAbs

Functional sequences without an intervening stop codon were selected for mAb production. Using previously defined approaches, the V_H_ and V_L_ amplicons were ligated to rhesus IgG1 backbone, CMV promoter regions, and a secretion sequence using overlapping PCR to form a linear amplicon (77, 79, 80). Ligation was confirmed by size on a gel, and successfully ligated sequences were then purified for DNA. Thus, each V_H_ and V_L_ formed a full-length expression cassette that co-transfected onto a monolayer of the human embryonic kidney cell line (293T) in six-well plates as per the manufacturer’s protocol (Expifectamine, Thermo). Supernatant containing recombinant IgG1 mAbs was collected in 3 days and concentrated (Millipore Ultra Centrifugal Spin Filter). This transfection supernatant was tested for rhesus IgG content and screened for ZIKV reactivity.

### Recombinant mAb production and purification

Isolation and expression of monoclonal antibodies were previously described (77). Briefly, V_H_ and V_L_ were cloned into modified pCDNA3.1/hygro plasmid vectors containing either (i) rhesus IgG1 constant region (GenBank: AY292507), CMV promoter, Bovine growth hormone (BGH), poly(A) tail, and IgG leader sequences; (ii) kappa (GenBank: AY292503.1) constant; and (iii) lambda (GenBank: MF989841.1) constant regions. V_H_ and V_L_ plasmids (0.05 mg/plasmid) were co-transfected into Expi293F cells (ExpiFectamine 293 Transfection Kit, Thermo) at 2.5 million cells/mL in 100-mL flasks in suspension, as per the manufacturer’s instructions. Supernatants from cell cultures incubating at 37°C were harvested 5 days later, and IgG mAbs were purified using Pierce Protein A Beads (Thermo), as per the manufacturer’s instructions. MAbs were buffer exchanged with 60 mL of sterile 1× PBS. Purified MAbs were tested by Western blotting and Coomassie blue staining to confirm the presence of a ~150-kDa IgG product. Protein concentration was assessed by NanoDrop (Thermo).

### Measurement of IgA and IgG concentration by ELISA

To assess the concentrations of IgG in transfection supernatants and IgA in plasma, we coated 384-well plates (Corning Life Sciences) overnight at 4°C with 3 µg/mL mouse anti-Rhesus IgA (clone: 9B9; Nonhuman Primate Reagent Resource) or 2 µg/mL goat anti-Rhesus IgG (Rockland) and blocked with 40 µL/well of superblock (4% whey and 15% normal goat serum in 0.5% PBS/Tween-20). Plasma samples were serially diluted starting at 1:100 with threefold dilutions up till 8 dilution points, and small-scale transfection (SSTx, 10 µL/well) supernatant was diluted starting at 1:50 with 10-fold dilution up till 4 dilution points (10 µL/well). Then, this was added to the plate. IgA antibodies were detected using Alpha Rhesus IgA 10F12 Biotin (1:1,000, Nonhuman Primate Reagent Resource) followed by HRP-conjugated streptavidin (1:10,000, Thermo). IgG antibodies were detected using mouse anti-macaque IgG HRP (1:10,000, Southern Biotech). Then, SureBlue (KPL) was added, and plates were read at 450 nm after addition of stop solution (KPL). Macaca mulatta dimeric IgA [b12rA1d] at 1 µg/mL with fourfold dilutions (Nonhuman Primate Reagent Resource) and Rhesus IgG1 CH22 antibody at 4 µg/mL with twofold dilutions were used as the standard and positive control, respectively. Negative control was block alone or cell culture media. Antibody concentrations were interpolated from the linear range standard curve, which was fit to a five-parameter sigmoidal curve (Molecular Devices SoftMax Pro 6.3 or BioTek GEN5 3.11 software). The limit of detection for IgA was 0.98 ng/mL, and for IgG ,it was 7.81 ng/mL. The quality control criterion was a <20% coefficient of variation between two replicates. IgA concentrations were inferred at a sample dilution of 1:300 or 1:24,300, and IgG concentrations were inferred at a dilution of 1:50. Superblock alone served as the negative control, and all samples and control were run in replicate. The Anti-rhesus IgA [10F12]-biotin antibody (Cat#PR-0126, RRID:AB_2819304), Anti-rhesus IgA [9B9] antibody (Cat#PR-9290, RRID:AB_2819305), and dimeric IgA [b12rA1d] (Cat#PR-1220, RRID:AB_2819329) were engineered and produced by the Nonhuman Primate Reagent Resource (NIH Nonhuman Primate Reagent Resource).

### Statistics

Mann-Whitney and Kruskal Wallis tests of significant differences were performed with GraphPad Prism 9.2.0, Hutcheson’s test of significant differences in diversity was performed with Microsoft Excel, and Fisher’s exact test was conducted using an online calculator (https://www.socscistatistics.com/tests/fisher/default2.aspx). Circos plots were made in R studio.

## Data Availability

The plasmablast variable region sequences from 363 single cells analyzed in this study are publicly available at the following Github repository (https://github.com/Dr-Tulika-Singh/Rhesus-ZIKV-Plasmablast-Seq).

## SUPPLEMENTAL MATERIAL

Supplemental material mbio.03160-23-s0001.docx Supplemental figures and tables.

ASM does not own the copyrights to Supplemental Material that may be linked to, or accessed through, an article. The authors have granted ASM a non-exclusive, world-wide license to publish the Supplemental Material files. Please contact the corresponding author directly for reuse.
