# Supplementary material for: Prior dengue virus serotype 3 infection modulates subsequent plasmablast responses to Zika virus infection in rhesus macaques
Source: mBio. 2024 Feb 13;15(3):e03160-23. doi: 10.1128/mbio.03160-23 (PMC10936420; doi:10.1128/mbio.03160-23)
Supplement: Supplemental material — Supplemental figures and tables. [file mbio.03160-23-s0001.docx]

**Supplemental Materials**

**Prior dengue virus serotype 3 infection modulates subsequent plasmablast responses to Zika virus infection in rhesus macaques**

Tulika Singh, Itzayana G. Miller, Sravani Venkatayogi, Helen Webster, Holly J. Heimsath, Josh A. Eudailey, Dawn M. Dudley, Amit Kumar, Riley J. Mangan, Amelia Thein, Matthew T. Aliota, Christina M. Newman, Mariel S. Mohns, Meghan E. Breitbach, Madison Berry, Thomas C. Friedrich, Kevin Wiehe, David H. O’Connor, and Sallie R. Permar


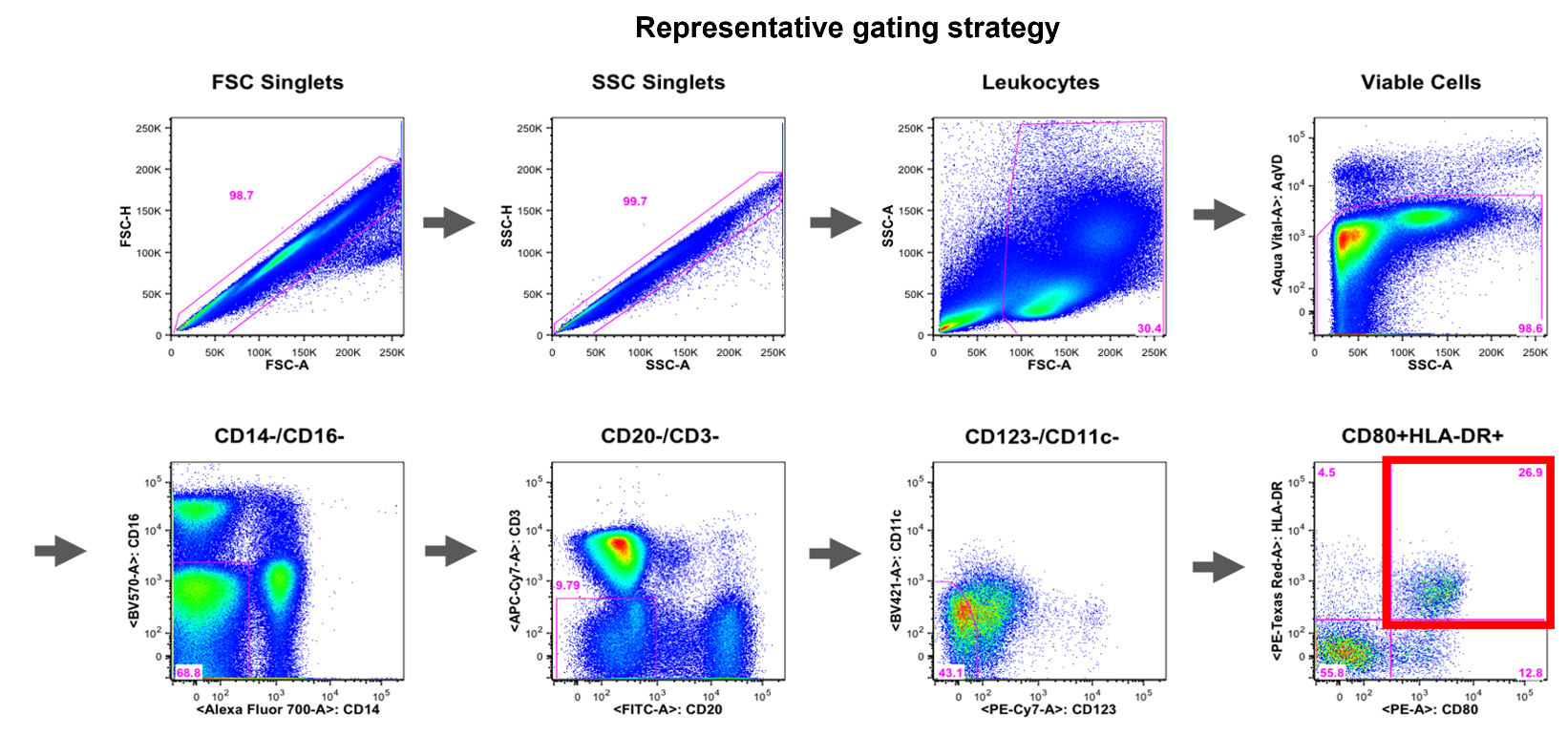


**Figure S1**. **Representative negative selection gating strategy for activated PBs from Rh** **826226 at 7 days post primary ZIKV challenge.** The subset of singlets and leukocytes was determined by gating on size and granularity of stained PBMCs. Followed by gating on live cells and then selection of cells within the CD14-/CD16-/CD20-/CD3-/CD123-/CD11c-/CD80+/HLADR+ population, as previously described for rhesus activated PBs (45). PBMCs were cryopreserved for use in this study.


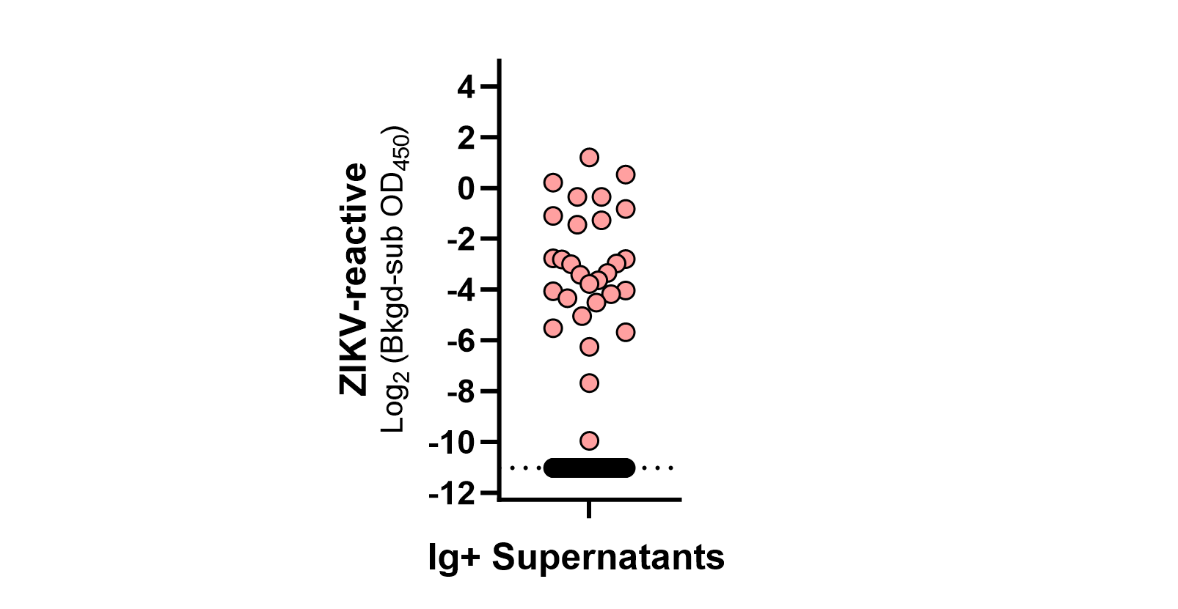


**Figure S2. Screen of ZIKV-reactivity with small-scale monoclonal antibody transfection supernatants derived from PB sequences.** V_H_ and V_L_ genes from sequenced PBs were ligated to rhesus IgG1 constant regions and co-transfected onto 293T cells to test the ZIKV-reactivity of the original PBs. All resulting immunoglobulin secreting supernatants were tested for Zika virion reactivity by ELISA. Magnitude of ZIKV-reactivity was assessed as optical density at 450nm (OD_450_) for each supernatant. The background of this ELISA was quantified as one standard deviation above the mean OD_450_ of a seronegative plasma diluted 1:1000 dilution on the day of the assay. Background subtracted OD_450_ are plotted and the supernatants determined as ZIKV-reactive are indicated in pink. PB derived supernatants were screened for each macaque and timepoint. Of the 177 supernatants tested, 7 contained duplicate sequences and were excluded from further analysis. One supernatant did not pass our quality control of reproducible replicates with <20% coefficient of variation. Thus, a total of 29 supernatants and their respective unique PBs were defined as ZIKV-reactive (17%; 29/169). For plotting on Log2 scale, the background subtracted value of 0 was set to 0.00048, where a dotted line indicates the level of background signal for this experiment. See Table 1.


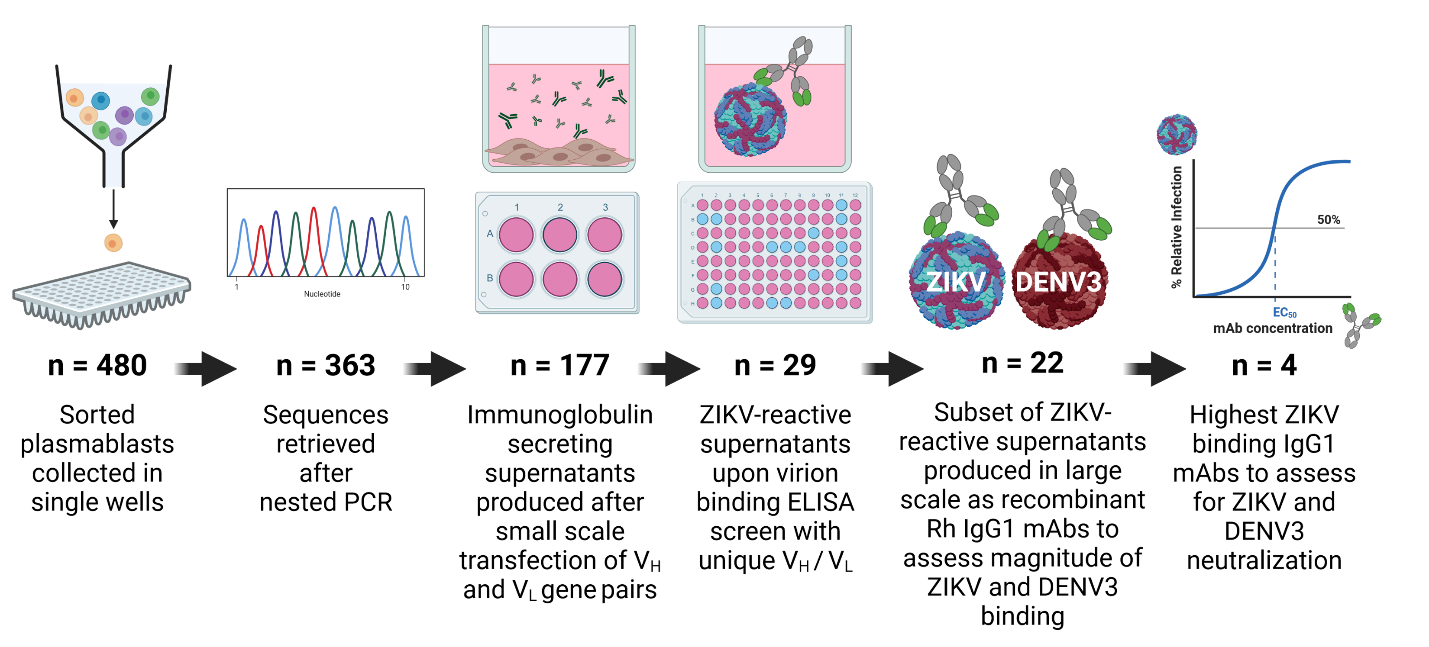


**Figure S3. Flow chart of the experimental process in this study with key indicators such as number of cells, sequences, transfection supernatants, and recombinant mAbs at each stage.** In total, 480 PBs were collected as single cell-per-well and attempted for nested PCR amplification of immunoglobulin variable region heavy and light chains. Then, 363 sequences were retrieved and analyzed for immunoglobulin variable gene use, isotype, HCDR3 length, and somatic mutation. Each functional heavy and light chain sequence was attempted for ligation into a linear amplicon with promoter, rhesus IgG1 constant region backbone, and secretion sequence elements for recombinant monoclonal antibody synthesis by transfection onto cells. If the heavy chain could be incorporated into an amplicon but the corresponding light chain could not, then paired transfection was not possible. In total, 177 small scale transfections within individual wells of a 6-well plate yielded any detectable rhesus IgG. These 177 supernatants containing varying levels of rhesus IgG were screened for ZIKV reactivity, and 29 were found to be ZIKV-reactive. Subsequently, we selected 22 clones for large scale recombinant monoclonal antibody production and tested the strength of ZIKV and DENV-3 binding. Of these, we chose the four highest binders for testing the strength of ZIKV and DENV-3 neutralization.

**Table S1. Closest human immunoglobulin variable V_H_ (top) and V_L_ (bottom) genes used in ZIKV-reactive PBs.**

|  | **ZIKV** | | **ZIKV-ZIKV** | | **DENV3-ZIKV** | | |  | | | |  |  |  | |
| --- | --- | --- | --- | --- | --- | --- | --- | --- | --- | --- | --- | --- | --- | --- | --- |
| **Rhesus VH gene** | **Rh826226** | **Rh912116** | **Rh826226** | **Rh912116** | | **Rh321142** | **Rh850585** | ***Zika binding*** | **Human VH gene** | **ID%** | ***Identified in other studies with human samples*** | | | |  |
| **IGHV1-c** | 0 | 0.027 | 0 | 0 | | 0.034 | 0 | 0.034 | **IGHV1-8** | 90.54 | **Parameswaran** et al., 2013 (DENV plasmablasts) | | | |  |
| **IGHV2-a/b** | 0.016 | 0.081 | 0 | 0.017 | | 0.017 | 0.017 | 0.069 | **IGHV2-70** | 93-94 | **Stettler** et al., 2016 (ZIKV-reactive); **Sapparapu** et al., 2016 (ZIKV-reactive); **Parameswaran** et al., 2013 (DENV plasmablasts) | | | |  |
| **IGHV2-d** | 0 | 0.027 | 0 | 0.034 | | 0 | 0 | 0.034 | **IGHV2-5** | 95.24 | **Appanna** et al., 2016 (DENV recE binding); **Parameswaran** et al., 2013 (DENV plasmablasts); **Wec** et al., 2020 (Yellow fever virus neutralizing and envelope protein binding) | | | |  |
| **IGHV3-aa** | 0 | 0 | 0 | 0 | | 0.017 | 0 | 0.034 | **IGHV3-13** | 92.15 |  | | | |  |
| **IGHV3-ag** | 0 | 0 | 0 | 0 | | 0.034 | 0.050 | 0.034 | **IGHV3-66** | 89.86 | ZIKV and DENV3 neutralizing in this study; **Stettler** et al., 2016 (ZIKV-reactive); **Parameswaran** et al., 2013 (DENV plasmablasts); **Wec** et al., 2020 (Yellow fever virus envelope protein binding) | | | |  |
| **IGHV3-d** | 0.016 | 0 | 0.030 | 0 | | 0.103 | 0 | 0.069 | **IGHV3-23** | 91.22 | **Singh** et al., 2022 (ZIKV/DENV cross-reactive); **Appanna** et al., 2016 (DENV recE binding); **Rogers** et al., 2017 (ZIKV EDIII binding, ZIKV neutralizing and convergent evolution to this gene in plasmablasts); **Robbiani** et al., 2017 (ZIKV EDIII binding, ZIKV-neutralizing, and convergent evolution to this gene in plasmablasts); **Stettler** et al., 2016 (ZIKV-reactive); **Sapparapu** et al., 2016 (ZIKV-reactive); **Parameswaran** et al., 2013 (DENV plasmablasts); **Wec** et al., 2020 (Yellow fever virus neutralizing and envelope protein binding) | | | |  |
| **IGHV3-h/j** | 0.016 | 0 | 0.119 | 0.034 | | 0.034 | 0 | 0.069 | **IGHV3-49** | 84-92 | **Appanna** et al., 2016 (DENV binding); **Stettler** et al., 2016 (ZIKV-reactive); **Parameswaran** et al., 2013 (DENV plasmablasts); **Wec** et al., 2020 (Yellow fever virus envelope protein binding) | | | |  |
| **IGHV4-b** | 0.210 | 0.027 | 0.030 | 0 | | 0 | 0.033 | 0.034 | **IGHV4-59** | 94.59 | **Appanna** et al., 2016 (DENV recE binding); **Stettler** et al., 2016 (ZIKV-reactive); **Parameswaran** et al., 2013 (DENV plasmablasts); **Wec** et al., 2020 (Yellow fever virus neutralizing and envelope protein binding) | | | |  |
| **IGHV4-e/g/j** | 0.113 | 0.216 | 0.224 | 0.254 | | 0.259 | 0.333 | 0.276 | **IGHV4-4** | 92-92 | **Appanna** et al., 2016 (DENV binding); **Parameswaran** et al., 2013 (DENV plasmablasts); **Wec** et al., 2020 (Yellow fever virus neutralizing and envelope protein binding) | | | |  |
| **IGHV4-n/f** | 0.274 | 0.135 | 0.373 | 0.271 | | 0.241 | 0.200 | 0.345 | **IGHV4-38-2** | 91-93 |  | | | |  |
| **Rhesus VL gene** | **Rh826226** | **Rh912116** | **Rh826226** | **Rh912116** | | **Rh321142** | **Rh850585** | ***Zika binding*** | **Human VL gene** | **ID%** | ***Identified in other studies with human samples*** | | | |  |
| **IGKV1-e** | 0.015 | 0.077 | 0.074 | 0.055 | | 0.103 | 0.067 | 0.103 | **IGKV1-5** | 93.0314 | **Rogers** et al., 2017 (ZIKV EDIII binding and convergent evolution to this gene in plasmablasts); **Robbiani** et al., 2017 (ZIKV EDIII binding and convergent evolution to this gene in plasmablasts); **Sapparapu** et al., 2016 (ZIKV-reactive); **Wec** et al., 2020 (Yellow fever virus envelope protein binding) | | | |  |
| **IGKV1-g** | 0.046 | 0.051 | 0.059 | 0.027 | | 0.103 | 0.017 | 0.069 | **IGKV1-12** | 94.4251 | **Sapparapu** et al., 2016 (ZIKV-reactive) | | | |  |
| **IGKV2-o** | 0 | 0 | 0 | 0 | | 0.034 | 0.017 | 0.034 | **IGKV2-28** | 92.053 | **Singh** et al., 2022 (ZIKV/DENV cross-reactive) | | | |  |
| **IGKV3-a/f** | 0.046 | 0.051 | 0.088 | 0 | | 0 | 0 | 0.069 | **IGKV3-20** | 94 | **Singh** et al., 2022 (ZIKV-binding); **Sapparapu** et al., 2016 (ZIKV-reactive) | | | |  |
| **IGKV4-a** | 0 | 0 | 0.029 | 0 | | 0 | 0.017 | 0.034 | **IGKV4-1** | 95.082 |  | | | |  |
| **IGLV10-a** | 0.015 | 0 | 0.029 | 0 | | 0.017 | 0 | 0.034 | **IGLV10-54** | 95.6081 |  | | | |  |
| **IGLV11-a** | 0 | 0 | 0.029 | 0 | | 0.034 | 0.033 | 0.069 | **IGLV5-37** | 78.8462 |  | | | |  |
| **IGLV1-e** | 0 | 0.051 | 0.015 | 0.014 | | 0.034 | 0 | 0.069 | **IGLV1-51** | 93.4783 | **Sapparapu** et al., 2016 (ZIKV-reactive); **Wec** et al., 2020 (Yellow fever virus envelope protein binding) | | | |  |
| **IGLV1-f** | 0.015 | 0 | 0 | 0.027 | | 0 | 0.017 | 0.034 | **IGLV1-47** | 94.2568 |  | | | |  |
| **IGLV1-i** | 0 | 0.026 | 0 | 0 | | 0 | 0.017 | 0.034 | **IGLV1-40** | 90.9699 |  | | | |  |
| **IGLV2-c** | 0 | 0 | 0.029 | 0 | | 0.017 | 0 | 0.034 | **IGLV2-23** | 92.9293 |  | | | |  |
| **IGLV2-e** | 0 | 0.026 | 0 | 0 | | 0 | 0 | 0.034 | **IGLV2-18** | 87.8788 |  | | | |  |
| **IGLV2-f** | 0.108 | 0.026 | 0.029 | 0.041 | | 0 | 0.01666667 | 0.034 | **IGLV2-11** | 95.9596 |  | | | |  |
| **IGLV2-j** | 0.046 | 0.026 | 0.074 | 0.082 | | 0.052 | 0.100 | 0.034 | **IGLV3-27** | 92.9078 |  | | | |  |
| **IGLV3-j** | 0.015 | 0.077 | 0 | 0.055 | | 0.069 | 0.017 | 0.034 | **IGLV3-21** | 92.4138 | **Singh** et al., 2022 (ZIKV-binding) | | | |  |
| **IGLV3-l** | 0.015 | 0 | 0.015 | 0 | | 0 | 0 | 0.034 | **IGLV3-1** | 85.906 |  | | | |  |
| **IGLV5-b** | 0.354 | 0 | 0 | 0 | | 0 | 0.017 | 0.138 | **IGLV5-39** | 91.6667 |  | | | |  |
| **IGLV6-c** | 0 | 0.026 | 0 | 0 | | 0 | 0.067 | 0.034 | **IGLV6-57** | 89.9666 |  | | | |  |
| **IGLV7-a/c** | 0.015 | 0 | 0 | 0.027 | | 0 | 0 | 0.069 | **IGLV7-43** | 91-92 |  | | | |  |


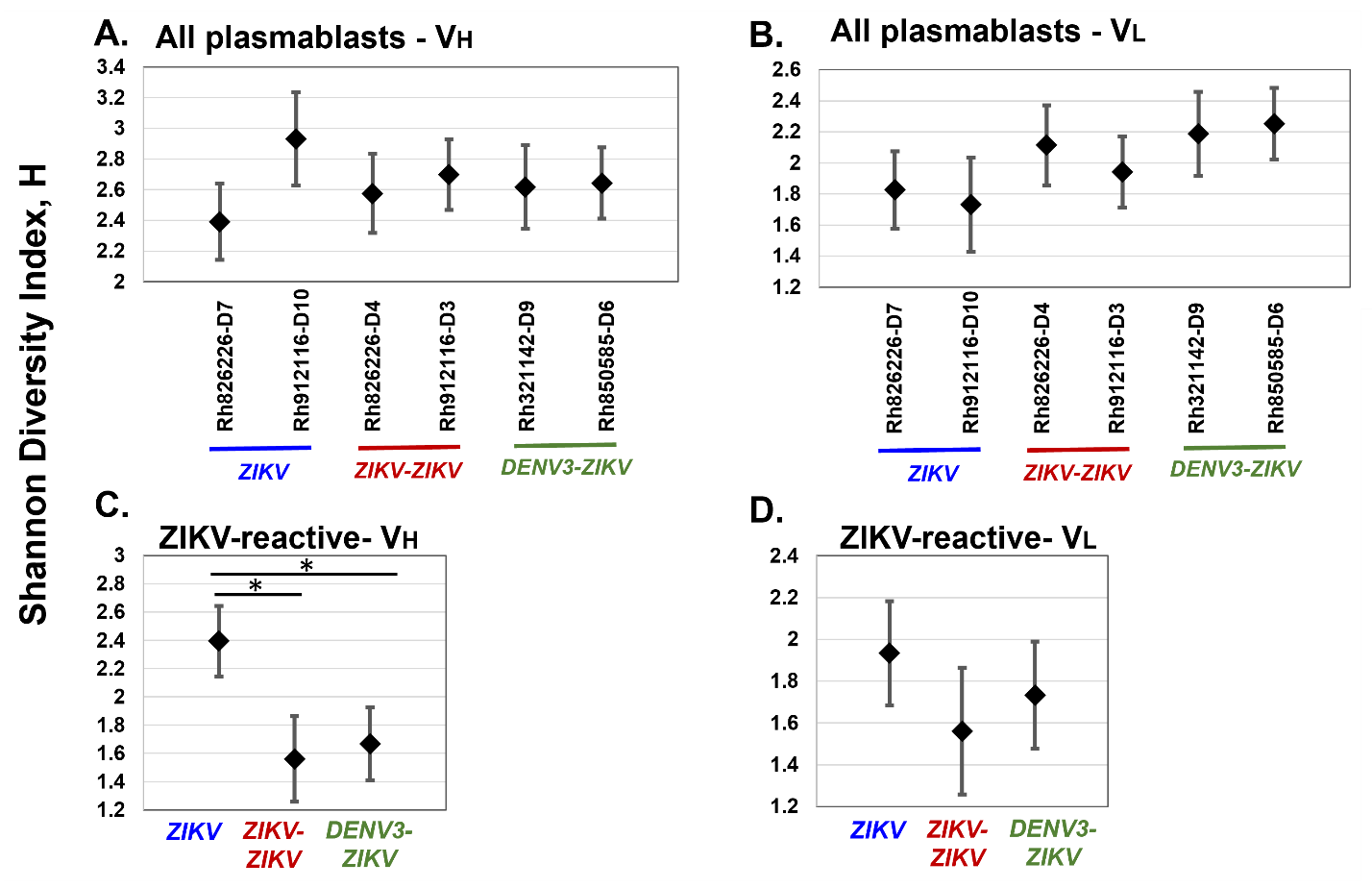


**Figure S3. Shannon Diversity Index of variable genes used in PB response to ZIKV-challenge in the V_H_ (A) and V_L_ of *all* PBs, versus the V_H_ (C) and V_L_ (D) of the *ZIKV-reactive* PBs subset.** Shannon diversity index (H) is a metric to define the number of distinct species in a given population, the higher the value the more distinct species present. B cell clones within each PB compartment were evaluated for diversity based on their V_H_ or V_L_ gene identity, by each macaque or challenge group. The 95% confidence interval for each estimated Shannon diversity index is plotted and significant differences are denoted (*). Hutcheson t-test was applied to test for significant differences in H.


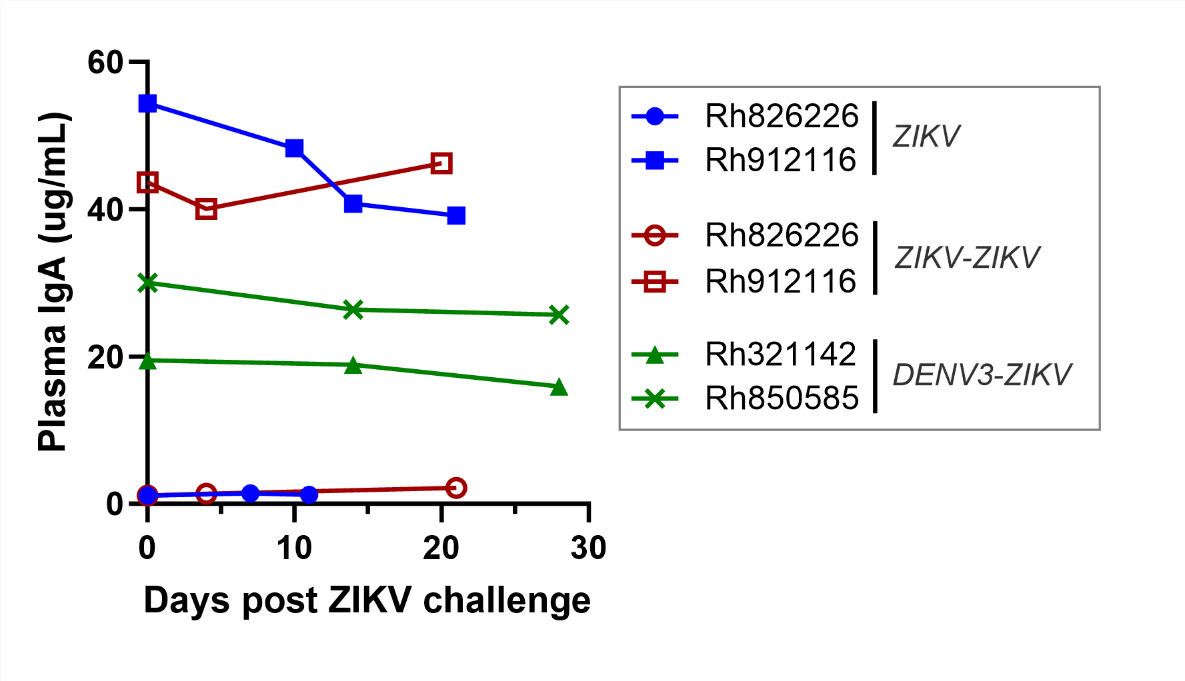


**Figure S4. Stable concentration of total IgA in plasma upon ZIKV challenge despite rise in IgA PBs.** Rhesus plasma was serially diluted, and concentration of IgA was detected via immunoglobulin-capture ELISA at an optical density at 450nm (OD_450_). The concentration of IgA in rhesus plasma was inferred from the OD_450_ at 1:300 plasma dilution, based on interpolation to a standard curve of purified rhesus IgA with known concentration. Colors indicate challenge group: Primary ZIKV challenge group (blue), secondary ZIKV-ZIKV group (red), and secondary DENV-3-ZIKV group (green).
